# Unveiling the Dynamic Mechanisms of Generative AI in English Language Learning: A Hybrid Study Based on fsQCA and System Dynamics

**DOI:** 10.3390/bs14111015

**Published:** 2024-10-31

**Authors:** Yang Zhang, Changqi Dong

**Affiliations:** 1 Faculty of Humanities & Social Sciences, Harbin Institute of Technology, Harbin 150001, China; zhangyanghit@hit.edu.cn; 2 School of Management, Harbin Institute of Technology, Harbin 150001, China

**Keywords:** English language learning, generative artificial intelligence, higher education, multi-dimensional mechanisms, qualitative comparative analysis, system dynamics

## Abstract

The burgeoning development of generative artificial intelligence (GenAI) has unleashed transformative potential in reshaping English language education. However, the complex interplay of learner, technology, pedagogy, and contextual factors that shape the effectiveness of GenAI-assisted language learning remains underexplored. This study employed a novel mixed-methods approach, integrating qualitative comparative analysis (QCA) and system dynamics (SD) modeling, to unravel the multi-dimensional, dynamic mechanisms underlying the impact of GenAI on English learning outcomes in higher education. Leveraging a sample of 33 English classes at the Harbin Institute of Technology, the QCA results revealed four distinct configurational paths to high and low learning effectiveness, highlighting the necessary and sufficient conditions for optimal GenAI integration. The SD simulation further captured the emergent, nonlinear feedback processes among learner attributes, human–computer interaction, pedagogical practices, and ethical considerations, shedding light on the temporal evolution of the GenAI-empowered language-learning ecosystem. The findings contribute to the theoretical advancement of intelligent language education by constructing an integrative framework encompassing learner, technology, pedagogy, and context dimensions. Practical implications are generated to guide the responsible design, implementation, and optimization of GenAI in English language education, paving the way for learner-centric, adaptive learning experiences in the intelligence era.

## 1. Introduction

The rapid advancement of artificial intelligence (AI) technologies, particularly in natural language processing, knowledge graphs, and deep learning, is accelerating their integration into the education sector. This integration has given rise to a novel ecosystem of AI-empowered education [1]. Generative artificial intelligence (GenAI), as a cutting-edge application of AI in education, offers unique advantages in natural language generation, automatic knowledge construction, and personalized recommendations. These capabilities have opened new avenues for achieving precision teaching and intelligent learning [2]. In the field of language education specifically, GenAI-driven applications such as intelligent writing assessment, adaptive language exercise generation, and immersive voice interaction have injected new vitality into traditional teaching and learning paradigms.

Against the backdrop of global higher education internationalization, English, as a universal academic language and intercultural communication tool, has assumed an increasingly prominent strategic position in talent cultivation [3]. Concurrently, traditional English teaching models are facing a series of challenges, including a surge in students’ personalized learning needs, insufficient intelligent teaching capabilities among educators, and low classroom interaction efficiency [4]. GenAI offers new perspectives for addressing these challenges. However, the question of how to deeply exploit the educational application potential of GenAI, promote its organic integration with English teaching, and ultimately achieve a transformative reconstruction of teaching and learning methods remains a theoretical issue and practical proposition urgently requiring exploration.

Existing research has preliminarily explored the application prospects of GenAI in language education from various perspectives, including technological characteristics [5], learning experience optimization [6], and teacher capacity development [7]. In the field of learning analytics, Farrelly and Baker (2023) proposed a learner-centered analytical framework, exploring GenAI’s personalized learning support mechanisms [8]. From an educational equity perspective, Holmes et al. (2022) systematically reviewed the ethical risks posed by GenAI, such as algorithmic bias and privacy protection, emphasizing the importance of value-oriented proactive governance [1]. While these explorations have laid an important foundation for the expanded application of GenAI in language education, they are subject to the following limitations: Firstly, there is a lack of systematic examination of the factors influencing the effectiveness of GenAI applications in the learning process, and research on the interaction mechanisms between cognitive, motivational, metacognitive, and other learning elements with intelligent technologies remains to be deepened [9]. Secondly, there is insufficient consideration of the long-term and dynamic nature of GenAI applications in language teaching, lacking a holistic grasp of the evolutionary trends and emergent patterns in intelligent teaching ecosystems [10].

Given the current state of research, this study focuses on GenAI-assisted English teaching in higher education as the research context. It adopts a mixed research paradigm combining Qualitative Comparative Analysis (QCA) and System Dynamics (SD) to explore the key element combinations affecting learning outcomes and their dynamic evolutionary patterns. On the one hand, case analysis based on QCA will employ necessary condition analysis and sufficient condition analysis to systematically examine how multidimensional factors such as learners, teachers, technology, and teaching interact and collaboratively shape learning outcomes. On the other hand, SD modeling and simulation will depict the dynamic evolutionary landscape of the GenAI-driven English learning ecosystem, revealing the dynamic feedback mechanisms between learning effects and key influencing factors. The innovative fusion of these two analytical paradigms is expected to achieve a research paradigm innovation from static to dynamic and from partial to holistic, providing new perspectives for understanding the generative logic and evolutionary patterns of GenAI-empowered language learning.

Overall, the academic contributions of this study are manifested in three aspects: Firstly, it constructs an integrative multidimensional impact analysis framework for GenAI-assisted English teaching, providing a systematic cognitive schema for expanding the theoretical connotations of intelligent language education. Secondly, it innovatively integrates QCA and SD analytical paradigms, revealing key influencing factor combinations through cross-case comparisons and grasping the evolution of intelligent teaching ecosystems through dynamic modeling, setting a paradigm for methodological innovation in intelligent education research. Thirdly, it generates a series of contextualized practical implications, offering normative guidance for optimizing GenAI educational application practices at the levels of curriculum design, teaching organization, and capacity building. The findings of this study not only contribute to innovating intelligent English teaching practices and enhancing the effectiveness of GenAI educational applications but also provide theoretical references for understanding the general rules and pathways of intelligent technology transforming education.

The structure of this paper is organized as follows: Section 2, based on a literature review, constructs a multidimensional impact mechanism analysis framework for GenAI-assisted English teaching. Section 3 details the research method design, focusing on the application steps of QCA and SD methods. Section 4 reports the empirical research results, concentrating on the key influencing factor combinations identified by QCA and the dynamic feedback mechanisms revealed by SD. Section 5, based on empirical findings, discusses in depth the theoretical mechanisms of GenAI empowering English teaching and distills practical implications. Section 6 concludes the paper and outlines future research directions.

## 2. Literature Review and Research Framework

### 2.1. Literature Review

The rapid development of AI technologies has led to increasing interest in the application of GenAI in the educational sphere. GenAI, with its unique advantages in natural language generation, personalized learning support, and real-time feedback, has paved new paths for intelligent language teaching [11]. Particularly in the context of higher education, GenAI has brought both new opportunities and challenges for enhancing English teaching quality and promoting learners’ language ability development [12].

Research on GenAI applications in education initially focused on exploring its technological characteristics and functional advantages. Wilson and Daugherty (2018) pointed out that AI can generate highly personalized and interactive language feedback by understanding natural language input, showing broad application prospects in grammar correction, writing assessment, and oral training [13]. Lee et al. (2020) further demonstrated GenAI’s unique advantages in providing personalized learning support, optimizing cognitive load, and promoting active learning, highlighting its enormous potential to empower language learning [14]. Empirical research by Cheng et al. (2021) showed that English writing instruction incorporating GenAI significantly enhanced learners’ writing motivation and problem-solving abilities [15]. These studies laid the theoretical foundation for GenAI applications in language teaching.

However, the impact mechanism of GenAI in educational applications is far from a simple linear effect at the technological level. It involves complex interactions among multiple elements, including learners, teachers, technology, teaching, and the environment. To systematically explore the influence mechanism of GenAI applications, researchers have begun to construct integrative analytical frameworks from multiple theoretical perspectives, including technology acceptance and use, learning effect evaluation, human–computer interaction, and educational equity [16].

In terms of technology acceptance and use, Davis’s (1989) Technology Acceptance Model (TAM) provided a theoretical basis for analyzing the behavioral intentions of teachers and students in accepting GenAI [17]. Key factors such as perceived usefulness, perceived ease of use, social influence, and facilitating conditions have been proven to significantly affect user acceptance [18]. Building on this, Venkatesh et al. (2003) proposed the Unified Theory of Acceptance and Use of Technology (UTAUT), further enriching the key variable dimensions influencing GenAI applications [19]. Scholars have applied the UTAUT model to examine Chinese university students’ willingness to accept intelligent learning assistants, finding that perceived usefulness and usage anxiety are core factors affecting acceptance willingness [20].

Learning effect evaluation is a key area in GenAI educational application research. A series of studies have focused on active learning and reflective learning supported by GenAI, exploring its shaping effects on learning processes and outcomes. These studies have highlighted the importance of considering various learning elements, such as cognitive, motivational, and meta-cognitive factors, in understanding the impact of GenAI on learning effectiveness. Hooshyar et al. (2020) developed a GenAI-based programming learning system, discovering that intelligent feedback and personalized learning path guidance significantly improved learners’ active learning levels and problem-solving performance, which are closely related to cognitive and motivational aspects of learning [21]. Moreover, GenAI-driven formative assessments can promote learners’ self-cognitive reflection, optimize metacognitive strategies, and achieve precise learning transfer, emphasizing the role of meta-cognitive elements in GenAI-supported learning [22]. Li et al. (2024) employed a quasi-experimental design, finding that GenAI-based collaborative writing environments can reduce learners’ writing anxiety and improve writing self-efficacy, further illustrating the interplay between motivational and cognitive factors in GenAI applications [23].

The introduction of GenAI has also profoundly impacted traditional classroom interaction and human–computer interaction patterns. Cognitive Load Theory (CLT) provides an important theoretical perspective for analyzing interaction design in GenAI applications. Kohnke (2024), based on CLT, analyzed cognitive load management strategies in human–machine collaborative flipped classrooms, emphasizing the importance of matching learning task complexity and optimizing information presentation methods to reduce learners’ extraneous cognitive load [24]. Zhonggen et al. (2019) used eye-tracking experiments to examine the effects of different human–computer interaction modes on learners’ cognitive load and learning engagement, finding that question–answer interaction and multi-channel feedback can effectively reduce cognitive load and enhance learning experiences [25]. Vaishnav (2024) further pointed out that human–computer interaction design tailored to different disciplinary characteristics and cognitive styles is key to leveraging the educational utility of GenAI [26].

Furthermore, the increasingly prominent ethical risks have raised researchers’ concerns about educational equity in GenAI applications. Aiken and Epstein (2000) were among the first to raise ethical issues in learning analytics, emphasizing the importance of data privacy protection in safeguarding student rights [27]. In recent years, with the prominence of issues such as algorithmic bias and information cocoons, researchers have begun to systematically examine the ethical dilemmas in GenAI applications [28]. McDonald et al. (2024), using typical ethical norms and policy texts as research objects, employed thematic analysis to distill three core principles of GenAI institutional ethics: protection of individual rights, transparency and accountability, and fairness and inclusivity [29]. Shin’s (2021) research further found that factors such as privacy policy presentation forms and algorithmic decision explainability have moderating effects on user trust and continued use intention [30]. These discussions provide normative bases for comprehensively examining the social and ethical risks of GenAI educational applications.

To further identify the research gaps and problems in the field of GenAI applications in language education, it is essential to review the literature from multiple perspectives, including learners, teachers, technology, teaching, management, and environment. Nasrullah and Al Wahyu (2024) conducted a systematic literature review on the application of ChatGPT in English language evaluation, highlighting the potential benefits and challenges of using GenAI for assessment purposes [31]. Ogunleye et al. (2024) provided a comprehensive review of GenAI applications for teaching and learning practice, emphasizing the need for more empirical studies on the effectiveness and implementation of GenAI in various educational contexts [32]. Tafazoli (2024) explored the potential of GenAI in democratizing English language education, discussing the implications for learners, teachers, and educational institutions [33]. These studies collectively point to the lack of systematic examination of the factors influencing the effectiveness of GenAI applications in the learning process, as well as the insufficient consideration of the long-term and dynamic nature of GenAI applications in language teaching.

In summary, the existing literature has preliminarily revealed the multifaceted influence mechanisms of GenAI applications in education from the technological, learning, interactive, and ethical dimensions. However, research findings from different perspectives still lack integration, and there remains insufficient systematic and dynamic examination of GenAI applications in language education contexts. Particularly in the specific context of English teaching, how various dimensional elements interact and dynamically evolve to progressively shape learning experiences, processes, and outcomes still lacks systematic analysis based on empirical data. Moreover, there is a need for more in-depth exploration of the interaction mechanisms between cognitive, motivational, meta-cognitive, and other learning elements with intelligent technologies, as well as the long-term and dynamic nature of GenAI applications in language teaching. In-depth exploration of this issue not only concerns the innovative development of intelligent language education theory but also relates to the direction and path of educational practice reform.

### 2.2. Research Framework

**Figure 1:**
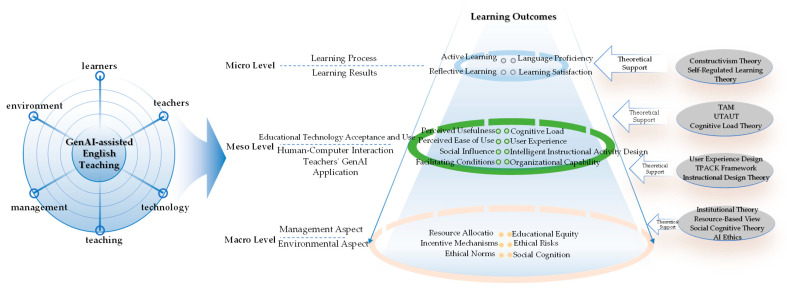
Multidimensional influence mechanism analysis framework for GenAI-assisted English teaching.

Based on a systematic review of existing theories, this study constructs an integrative analytical framework to explore the multidimensional dynamic influence mechanism of GenAI-assisted English teaching (as shown in Figure 1). This framework comprehensively considers six major dimensions, learners, teachers, technology, teaching, management, and environment, focusing on learning effect as the core variable, aiming to depict the internal operational mechanism of the intelligent English teaching ecosystem in a multi-level and dynamic manner.

At the micro level, the framework focuses on learning processes and outcomes, integrating key perspectives from the Technology Acceptance Model (TAM), Unified Theory of Acceptance and Use of Technology (UTAUT), and Cognitive Load Theory (CLT). The learning process emphasizes examining learners’ linguistic practices supported by GenAI from the perspectives of active learning and reflective learning, drawing on insights from Constructivism Theory and Self-Regulated Learning Theory. This process is influenced by individual differences in learners’ acceptance and use of GenAI tools, as conceptualized in TAM and UTAUT, which posit that factors such as perceived usefulness, perceived ease of use, social influence, and facilitating conditions are critical determinants of technology adoption. Additionally, the framework incorporates insights from CLT, recognizing that the cognitive load imposed by GenAI interfaces and interactions can significantly impact learners’ engagement and performance. Learning outcomes focus on English learning effectiveness as the core consideration, examined from dimensions such as language proficiency and learning satisfaction.

The meso level focuses on key factors affecting the efficacy of GenAI teaching applications, drawing on the Technological Pedagogical Content Knowledge (TPACK) Framework, Instructional Design Theory, and User Experience Design principles. The TPACK Framework emphasizes the importance of teachers’ integrated knowledge of technology, pedagogy, and content in designing and implementing effective technology-enhanced learning experiences. Instructional Design Theory provides guidance on creating optimal learning conditions by aligning learning objectives, instructional strategies, and assessment methods. User Experience Design principles highlight the critical role of usability, accessibility, and engagement in shaping learners’ interactions with GenAI tools. The framework thus considers teachers’ proficiency in leveraging GenAI to design and organize pedagogically sound and user-centered learning activities as a key factor influencing the success of GenAI-assisted English teaching.

The macro level examines the institutional environment and social context of the intelligent English teaching ecosystem, informed by Institutional Theory, Resource-Based View, Social Cognitive Theory, and AI Ethics frameworks. Institutional Theory emphasizes the role of regulatory, normative, and cultural–cognitive factors in shaping organizational behaviors and outcomes. The Resource-Based View highlights the importance of strategic resource allocation and management in sustaining competitive advantage and long-term performance. Social Cognitive Theory underscores the triadic reciprocal relationship between personal, behavioral, and environmental factors in shaping human functioning and learning. AI Ethics frameworks, such as the IEEE Ethically Aligned Design guidelines, provide principles and recommendations for ensuring the responsible and trustworthy development and deployment of AI systems. The framework thus considers institutional designs, resource allocation, social norms, and ethical considerations as critical components of the macro ecology of intelligent English teaching applications.

In integrating these theoretical perspectives, the framework seeks to leverage their complementary insights while minimizing potential contradictions or overlaps. For example, while TAM, UTAUT, and CLT offer different lenses on the micro-level factors influencing learner engagement with GenAI, they converge in highlighting the importance of considering both technological and cognitive aspects of the learning process. Similarly, the TPACK Framework, Instructional Design Theory, and User Experience Design principles, while focusing on distinct aspects of the meso-level factors shaping the efficacy of GenAI teaching applications, are interconnected in their emphasis on the need for pedagogically grounded, learner-centered, and contextually relevant design and implementation of educational technologies.

This study conducts empirical research guided by the above analytical framework, employing a mixed-methods approach that combines Qualitative Comparative Analysis and System Dynamics modeling. QCA enables a systematic examination of how key influencing factors and their combinations at micro, meso, and macro levels shape English learning effectiveness by identifying necessary and sufficient conditions for different learning outcomes. SD modeling and simulation, on the other hand, dynamically depict the evolutionary landscape of the English learning ecosystem, revealing dynamic feedback relationships and emergent mechanisms among various levels and elements, and providing insights into the long-term sustainability and systemic impacts of GenAI interventions.

The fusion of these qualitative and quantitative analytical paradigms is expected to achieve a comprehensive research paradigm innovation from static to dynamic and from partial to holistic, laying a solid foundation for constructing a theory of GenAI-driven English education transformation. The integrative framework, with its multi-level and multi-dimensional focus, serves as a conceptual anchor for this ambitious methodological integration, ensuring the coherence and consistency of the empirical investigation and theory building.

## 3. Mixed Research Methods for Analyzing Dynamic Mechanisms of GenAI in English Language Learning

### 3.1. Research Design for Dynamic Mechanism Analysis

#### 3.1.1. Mixed-Methods Approach

This study adopts a mixed-methods approach, combining QCA and SD simulation to investigate the role of GenAI in supporting higher education students’ learning. The mixed-methods design allows for a comprehensive examination of the complex interactions among technological, pedagogical, and contextual factors shaping the adoption and effectiveness of GenAI in educational settings [34]. The combination of QCA and SD is particularly suitable for this study as it enables the identification of key configurational conditions leading to successful learning outcomes (via QCA) and explores how these conditions dynamically interact and feedback over time (via SD) [35]. This approach aligns with calls in the educational technology field for more comprehensive and integrative research methodologies that transcend the limitations of traditional variable-oriented approaches [36].

To ensure the transparency and robustness of the mixed-methods approach, an explicit protocol for identifying, analyzing, and reconciling potential divergences between QCA and SD results has been established. In cases where discrepancies arise between the two methods, the following steps will be taken: (1) re-examination of the data and analysis procedures to identify potential sources of divergence; (2) consultation with domain experts to provide additional insights and interpretations; (3) conducting sensitivity analyses to assess the impact of different assumptions and parameters on the results; and (4) triangulating findings with other data sources or the existing literature to enhance the credibility of the integrated conclusions. By systematically addressing potential divergences, this study aims to maintain the validity and reliability of the mixed-methods approach.

QCA, as a configurational comparative method, is rooted in set theory and Boolean algebra [37]. It allows for the systematic analysis of multiple cases to identify necessary and sufficient conditions for an outcome of interest. In the context of this study, QCA will be employed to uncover combinations of technology acceptance, learning design, cognitive load, human–computer interaction, and ethical considerations associated with high levels of student learning effectiveness in GenAI-supported courses.

Conversely, SD is a modeling and simulation approach focused on the structure and dynamics of complex systems [38]. It represents systems as networks of interconnected feedback loops, capturing non-linear relationships and delays between variables [39]. By developing an SD model of GenAI adoption and learning processes, this study will elucidate the temporal dynamics and unintended consequences of policy interventions such as teacher training, technology support, and student preparation programs.

The mixed-methods design is implemented in an explanatory sequential manner [40], with the QCA phase informing the development of the SD model. Findings from QCA provide a theoretical foundation for identifying key variables, causal relationships, and feedback structures in the SD model. Specifically, the necessary and sufficient conditions identified by QCA will be translated into corresponding variables and causal links in the SD model. The configurational pathways uncovered by QCA will guide the specification of feedback loops and dynamic hypotheses in the SD model. Moreover, the consistency and coverage scores from QCA will inform the parameterization and calibration of the SD model, ensuring that the simulation results align with the empirical evidence. This sequential integration ensures that SD simulations are grounded in empirical evidence and theoretical insights, enhancing their validity and utility for policy analysis and decision-making.

#### 3.1.2. Rationale and Advantages

The rationale behind the mixed-methods approach lies in its ability to leverage the strengths of QCA and SD while mitigating their limitations. QCA focuses on configurational causality, allowing for the identification of complex patterns and contextual conditions that traditional regression-based approaches might overlook [41]. It is particularly suited for analyzing heterogeneity and equifinality in educational settings, where multiple pathways can lead to similar outcomes [42]. However, the static nature of QCA limits its ability to capture dynamic processes and feedback loops that unfold over time.

SD, on the other hand, excels at representing the dynamics and complexity of educational systems [43]. It can model the cumulative processes, delays, and feedback mechanisms that shape system behavior. By simulating different scenarios and policy interventions, SD provides insights into the long-term consequences and trade-offs of educational policies and practices. However, SD models are often criticized for lacking empirical grounding and the difficulty in validating their structure and parameters [44].

The integration of QCA and SD in this study addresses these limitations and offers several advantages. Firstly, QCA findings provide a robust empirical foundation for SD model development. The identified configurational conditions inform the selection of key variables and specification of causal relationships in the model. The process of integrating QCA results into the SD model involves the following steps: (1) mapping the necessary and sufficient conditions from QCA onto corresponding variables in the SD model; (2) translating the configurational pathways from QCA into feedback structures and dynamic hypotheses in the SD model; (3) using the consistency and coverage scores from QCA to calibrate the parameters and initial conditions of the SD model; and (4) iteratively refining the SD model structure and equations based on the insights gained from QCA. This systematic integration process enhances the validity and credibility of SD simulations, ensuring they are grounded in real-world evidence.

Secondly, the SD model extends insights gained from QCA by incorporating the temporal dimension and feedback processes. It allows for the exploration of how configurations identified by QCA evolve and interact over time, revealing the dynamic complexities of GenAI adoption and learning processes. This dynamic perspective is crucial for understanding the long-term impacts of educational policies and interventions, as well as potential unintended consequences and trade-offs.

Thirdly, the mixed-methods approach enhances the explanatory power and actionable insights of the research. QCA results provide a nuanced understanding of the contextual factors and causal mechanisms behind successful GenAI adoption and learning outcomes. SD simulations, in turn, offer a platform for testing and refining educational policies and strategies, enabling decision-makers to anticipate and manage the complex dynamics of educational change.

### 3.2. Qualitative Comparative Analysis (QCA)

#### 3.2.1. Case Selection: English Classes at HIT

This study selected 33 English teaching classes at the Harbin Institute of Technology (HIT) as case units for QCA. The case selection followed the principles of theoretical sampling, aiming to maximize variation and diversity in GenAI applications within higher education language-learning environments [45]. HIT, as one of China’s top universities in science and technology, exemplifies typical characteristics of technology-enhanced language teaching in its English courses, including diverse student populations, innovative teaching methods, and rich technological resources [46].

The selection of HIT’s English courses as the research subject is justified by several reasons. Firstly, HIT is at the forefront of educational technology application and innovation, with its English courses widely adopting various digital tools and platforms, including GenAI technologies [47]. This provides an ideal environment for examining the role of GenAI in supporting language learning. The GenAI tools and systems employed in the studied English classes encompass a range of capabilities and applications, reflecting the cutting-edge nature of HIT’s language education technology infrastructure. One prominent example is the use of intelligent tutoring systems (ITS) powered by GenAI algorithms. These systems, such as “SmartTutor” and “LanguageCoach”, provide personalized learning experiences by adapting to individual students’ proficiency levels, learning styles, and progress. They offer interactive exercises, real-time feedback, and targeted recommendations, enabling students to practice their language skills in a dynamic and engaging manner. The ITS platforms integrate natural language processing (NLP) and machine learning techniques to analyze students’ responses, identify strengths and weaknesses, and provide tailored support and guidance. Another key application of GenAI in HIT’s English classes is the use of conversational AI agents for language practice and assessment. Tools like “ChatMate” and “SpeakBuddy” leverage advanced dialogue systems and speech recognition technologies to provide students with immersive conversational experiences. These AI agents can engage in context-aware discussions, respond to students’ inquiries, and offer corrections and suggestions for improvement. By simulating realistic language interactions, these tools help students develop their communicative competence and build confidence in using English for authentic purposes. Moreover, the English classes at HIT also utilize GenAI-powered writing assistants and feedback systems. Applications such as “WriteMentor” and “EssayGPS” employ natural language generation and text analysis techniques to provide students with real-time feedback on their writing assignments. These tools can assess grammar, vocabulary usage, coherence, and style, offering suggestions for revision and improvement. They also provide personalized writing prompts, examples, and resources based on students’ specific needs and goals. By leveraging GenAI technologies, these writing assistants enable students to refine their writing skills and develop more effective strategies for self-expression and academic communication. In addition to these core GenAI applications, HIT’s English classes also incorporate various supplementary tools and resources that leverage GenAI capabilities. For instance, intelligent content recommendation systems curate and suggest personalized learning materials based on students’ interests, proficiency levels, and learning objectives. Adaptive vocabulary trainers and spaced repetition systems help students efficiently acquire and retain new words and phrases. GenAI-powered pronunciation coaching tools provide real-time feedback and visualization of students’ speech patterns, helping them improve their articulation and intonation. By providing these concrete details on the specific GenAI tools and systems used in the studied English classes, we aim to enhance the contextual richness and practical relevance of our research. This information enables readers to better understand the nature and capabilities of the GenAI technologies employed, assess their potential impact on language-learning processes and outcomes, and draw more meaningful comparisons with other GenAI implementations in educational settings.

Secondly, HIT’s English courses exhibit significant variations in instructional design, teacher–student interaction, and learning assessment, allowing for the exploration of how different configurational conditions influence learning effectiveness under GenAI support [48]. The studied classes encompass a spectrum of pedagogical approaches, ranging from traditional teacher-led instruction to more student-centered and collaborative learning models. Some classes emphasize explicit language instruction and drill-based practice, while others prioritize communicative tasks and project-based learning. The role of teachers also varies across classes, with some acting primarily as facilitators and guides, while others take on more directive and evaluative roles. Assessment practices range from traditional paper-based exams to performance-based assessments and portfolios. This diversity in instructional design, teacher–student interaction, and assessment allows for a nuanced examination of how GenAI technologies intersect with different pedagogical factors to shape learning effectiveness. By considering the interplay between GenAI tools and various instructional configurations, our study provides insights into the optimal conditions for leveraging GenAI to support language learning. This information is crucial for educators and researchers seeking to design and implement effective GenAI-enhanced language programs that align with specific pedagogical goals and contexts.

Lastly, the diversity in students’ learning backgrounds, motivations, and abilities is crucial for understanding GenAI applications across different learner groups. The English classes at HIT comprise students from various academic disciplines, including engineering, science, humanities, and social sciences. They also represent a range of English proficiency levels, from beginners to advanced learners. Some students are highly motivated to improve their language skills for academic or professional purposes, while others may have more general communicative goals. This learner diversity allows for an investigation of how GenAI tools and systems can be adapted and optimized to meet the needs and preferences of different student populations. By considering the varied learner characteristics and backgrounds, our study provides insights into the potential of GenAI technologies to personalize and differentiate language instruction. The detailed information on the specific GenAI tools and their capabilities, as described earlier, helps illustrate how these technologies can be leveraged to address individual learner differences. For instance, intelligent tutoring systems can adapt to students’ proficiency levels and provide targeted feedback and support, while conversational AI agents can offer personalized language practice opportunities tailored to students’ interests and goals. By examining the impact of GenAI across diverse learner groups, our study contributes to a more comprehensive understanding of how these technologies can promote equitable and inclusive language education.

The selection of the 33 English teaching classes at HIT followed a multi-stage process to ensure a diverse and representative sample. First, a comprehensive review of HIT’s English course offerings was conducted, considering factors such as proficiency levels, course types, and teaching modes. Second, consultations were held with language department administrators and instructors to identify classes that incorporated GenAI technologies in their teaching and learning practices. Third, a purposive sampling approach was employed to select classes that represented a range of language-learning contexts and GenAI applications, with attention to achieving a balance across key dimensions such as proficiency levels, course types, and teaching modes.

Prior to the commencement of this study, research ethics approval was obtained from the Institutional Review Board (IRB) at the Faculty of Humanities & Social Sciences at the Harbin Institute of Technology. The IRB reviewed the study protocol, including the informed consent procedures, to ensure that this research was conducted in accordance with ethical guidelines and regulations.

Following class selection, the instructors of the selected classes were invited to participate in this study. Informed consent was obtained from both instructors and students. Due to the fact that this study mainly used a survey questionnaire, which did not involve sensitive data, and the questionnaire was distributed to all classes taught by the author in English courses, the informed consent of the participants was obtained through the class monitor. There were many class members involved, and some doctoral students were not on campus. Therefore, after collecting the consent of all participants, the class monitor signed on their behalf. This approach was approved by the IRB, as it was deemed appropriate given the nature of this study and the logistical constraints in obtaining individual signatures from all participants. It is important to note that the participants’ decision to take part in the study was entirely voluntary, and they were free to withdraw at any time without any consequences. The researchers ensured that the participants were fully informed about the purpose, procedures, and potential risks and benefits of this study before obtaining their consent. The anonymity and confidentiality of the participants’ data were also strictly protected throughout the research process.

This multi-stage selection process, coupled with the rigorous informed consent procedures, ensured that the 33 English teaching classes included in our study reflected the diversity of language-learning experiences and GenAI implementations within HIT while also adhering to ethical principles of voluntary participation and informed consent.

Among the 33 English teaching classes, each class is considered a unique case unit. These classes cover different English proficiency levels (e.g., elementary, intermediate, and advanced), course types (e.g., English listening and speaking, reading, and writing), and teaching modes (e.g., face-to-face, online, and hybrid). By including cases across these dimensions, this research can comprehensively capture the application and effects of GenAI in diverse language-learning contexts. This approach allows for a more robust and generalizable understanding of how GenAI technologies can be integrated into various language course formats and delivery modes.

For instance, the inclusion of classes at different proficiency levels enables an examination of how GenAI tools can support language learning at various stages of development, from foundational skills to more advanced communicative competencies. The consideration of different course types allows for an exploration of how GenAI technologies can be adapted to support specific language skills and subskills, such as listening comprehension, oral production, reading strategies, and writing processes. The inclusion of diverse teaching modes provides insights into how GenAI tools can be effectively integrated into both traditional classroom settings and online or hybrid learning environments, addressing the unique challenges and opportunities of each delivery format.

By providing these specific details on the nature and capabilities of the GenAI technologies employed in the studied English classes, as well as the diverse pedagogical and learner factors considered, we aim to enhance the contextual richness, practical relevance, and generalizability of our research. This information enables readers to better assess the applicability of our findings to other GenAI implementations in language education and to make informed decisions about the design and deployment of GenAI technologies in their own teaching and learning contexts.

#### 3.2.2. Condition and Outcome Variables

Based on the aforementioned theoretical framework, the following condition and outcome variables were identified:

Condition variables:

(a) Technology acceptance (TA): the degree of acceptance and use of GenAI tools by students and teachers, including perceived usefulness, perceived ease of use, intention to use, and actual usage behavior [49].
(b) Learning design quality (LDQ): the quality of GenAI-supported English courses in terms of learning objectives, content organization, activity design, and assessment methods [50].
(c) Cognitive load level (CLL): the level of cognitive load experienced by students when using GenAI tools for language learning, including intrinsic and extraneous cognitive load [51].
(d) Human–computer interaction quality (HIQ): the quality of GenAI tools in terms of interface design, navigation structure, feedback mechanisms, and user control [52].
(e) Ethical considerations (EC): considerations in the application of GenAI in English courses regarding privacy protection, fairness, transparency, and accountability [53].

Outcome variable: learning effectiveness (LE)—students’ learning outcomes in GenAI-supported English courses, including indicators such as language proficiency improvement, learning satisfaction, engagement, and motivation [54].

The selection of these variables is grounded in extensive research, covering areas such as educational technology acceptance and use, learning design, cognitive load, human–computer interaction, and educational ethics. By incorporating these variables into QCA, this study can systematically explore their complex interactions and identify key configurational conditions leading to high learning effectiveness.

#### 3.2.3. Data Collection and Calibration

To obtain the data required for QCA, multiple data collection methods were employed. First, the researchers developed a survey questionnaire to measure students’ and teachers’ perceptions and attitudes regarding technology acceptance, cognitive load, and learning effectiveness. The questionnaire design drew on validated scales from relevant fields, such as the Technology Acceptance Model scale [55] and the Learning Engagement Scale [53]. The survey questionnaire consisted of several sections, each targeting specific constructs of interest.

In the technology acceptance section, questions were adapted from the Technology Acceptance Model (TAM) scale [55], which measures users’ perceived usefulness, perceived ease of use, and intention to use technology. For example, students were asked to rate their agreement with statements such as “Using GenAI tools enhances my language learning experience” and “I find GenAI tools easy to use for language learning tasks”. Similarly, teachers were asked about their perceptions of the usefulness and ease of use of GenAI tools in their teaching practices.

The cognitive load section of the questionnaire was based on the Cognitive Load Scale, which assesses the mental effort and difficulty experienced by learners during tasks. Students were asked to rate statements such as “Using GenAI tools for language learning tasks requires a lot of mental effort” and “I find it difficult to understand and use the feedback provided by GenAI tools”. These questions aimed to capture the cognitive load imposed by GenAI technologies on learners.

To measure learning effectiveness, the questionnaire incorporated items from the Learning Engagement Scale [56], which evaluates learners’ behavioral, emotional, and cognitive engagement in learning activities. Students were asked to rate their agreement with statements such as “I actively participate in language learning activities using GenAI tools” and “I feel motivated and interested when using GenAI tools for language learning”. These questions sought to assess the impact of GenAI technologies on students’ engagement and learning outcomes.

To ensure the reliability and consistency of the survey data, the questionnaire was pilot-tested, translated, and back-translated, and administered in a standardized manner with clear instructions and emphasis on anonymity and confidentiality.

In addition to the survey questionnaire, the researchers conducted systematic textual analysis of English course syllabi, learning materials, and assessment schemes to evaluate indicators of learning design quality and ethical considerations. A coding scheme was developed based on relevant frameworks, such as the Quality Matters rubric for online course design and the Ethical Framework for AI in Education.

To ensure the reliability and consistency of the textual analysis, a detailed coding manual was developed, providing clear definitions and examples for each category and subcategory. A team of trained coders independently coded a subset of the materials, and inter-rater reliability was assessed using Cohen’s kappa. Discrepancies were discussed and resolved through consensus, and the coding manual was refined based on the feedback. Regular calibration sessions were conducted throughout the coding process to maintain consistency and address any emerging issues.

Lastly, the researchers performed heuristic evaluations and usability tests on GenAI tools to assess human–computer interaction quality. A team of usability experts independently evaluated the GenAI tools used in the English classes using Nielsen’s 10 usability heuristics, and a sample of students and teachers participated in usability testing sessions, where they were asked to perform specific tasks using the GenAI tools while thinking aloud.

To ensure the reliability and validity of the heuristic evaluations and usability tests, multiple evaluators and test participants were used, and the findings were triangulated with other data sources, such as user feedback and system logs.

Following data collection, the researchers calibrated the raw data, transforming these into membership scores suitable for QCA. The calibration process followed Ragin’s [37] direct calibration method, determining full membership (1), crossover point (0.5), and full non-membership (0) thresholds for each variable based on theoretical knowledge and empirical distribution. The calibration process was conducted by a team of researchers with expertise in QCA and the substantive domain. To ensure the reliability and consistency of the calibration process, the researchers independently calibrated a subset of the data and compared their results to assess inter-rater reliability. Discrepancies were discussed and resolved through consensus, and the calibration rules were refined based on the feedback. Sensitivity analyses were conducted to examine the robustness of the findings to different calibration thresholds and methods, and the calibrated data were reviewed by external experts in QCA and the substantive domain to validate the appropriateness and credibility of the calibration decisions.

By employing multiple data collection methods, ensuring the reliability and consistency of the data through rigorous measures, and following a transparent and systematic calibration process, this study aimed to enhance the methodological rigor and trustworthiness of the QCA findings.

#### 3.2.4. Truth Table Analysis

After data calibration, the researchers conducted truth table analysis using fs/QCA 3.0 software [41]. The truth table lists all possible condition combinations and their corresponding case distribution and consistency scores. Consistency scores measure the subset relationship between a condition combination and the outcome, i.e., the degree to which cases in that combination have high outcome membership [57].

To identify sufficient conditions, the researchers set consistency and frequency thresholds. The consistency threshold ensures that extracted condition combinations have sufficient empirical evidence for the outcome, while the frequency threshold excludes the influence of accidental or anomalous cases [58]. In this study, the consistency threshold was set at 0.8, and the frequency threshold at 2. Condition combinations exceeding these thresholds were considered sufficient conditions for high learning effectiveness.

Based on the truth table analysis, the researchers further applied Boolean minimization algorithms to identify key condition combinations leading to high learning effectiveness. The minimization process, through logical minimization, removes redundant elements from condition combinations to obtain the most parsimonious sufficient condition expressions [35]. These expressions reveal complex causal relationships among different condition variables, demonstrating multiple pathways to achieving high learning effectiveness.

### 3.3. System Dynamics Simulation

#### 3.3.1. Causal Loop Diagram Construction

Building upon the key conditions and outcome variables identified through QCA, this study constructed a comprehensive and complex Causal Loop Diagram (CLD) to depict the dynamic feedback structures of GenAI applications in higher education language learning. The CLD construction process integrated multiple theoretical perspectives, including the Technology Acceptance Model [59], Teaching Presence Theory [51], Cognitive Load Theory [60], Human–Computer Interaction Theory [61], and Educational Equity and Ethics Frameworks [62]. It also incorporated multidimensional elements such as learners, teachers, technology, teaching, management, and environment to ensure model comprehensiveness and theoretical depth.

The overall CLD of the GenAI-supported higher education language-learning system presents a complex adaptive system comprising six main subsystems: learner, teacher, GenAI technology, instructional design, management decision, and social environment. These subsystems interact through intricate causal links and feedback loops, collectively shaping the dynamic processes and outcomes of GenAI-supported language learning. This systematic thinking approach enables us to transcend simple linear causality and delve into the system’s emergent and self-organizing properties.

In the learner subsystem, individual factors such as learning motivation, metacognitive strategies, and autonomous learning abilities form positive feedback loops (R1, R2) with language-learning engagement and learning outcomes, driving learners’ positive adaptation and efficient learning in the GenAI environment. Concurrently, the negative feedback relationship between cognitive load level and learning engagement (B1) reflects the balancing mechanism of load optimization in the learning process.

The teacher subsystem emphasizes the profound impact of teachers’ willingness to adopt and ability to implement GenAI tools on teaching effectiveness. This subsystem’s design is inspired by innovation diffusion theory, reflected in the positive feedback loop (R3) where improved teaching effectiveness incentivizes further GenAI adoption by teachers. However, the emergence of ethical risks may inhibit teachers’ usage behavior (B2), a negative feedback mechanism reflecting the core position of technology ethics in educational innovation, echoing Floridi’s discourse on digital ethics. Furthermore, the positive feedback loop between teachers’ GenAI literacy and student learning experience (R4) highlights the importance of enhancing teacher capabilities in promoting GenAI applications.

The GenAI technology subsystem precisely depicts the dynamic relationship between technological characteristics and system performance. This subsystem’s design integrates Davis’s [59] Technology Acceptance Model and the Information System Success Model, manifesting as positive feedback loops (R5, R6) where perceived ease of use and usefulness influence user satisfaction and continued use intention, thereby affecting GenAI adoption rates. The improvement in technological maturity drives enhancements in system reliability and interaction quality (R7), further elevating teaching and learning experiences. This mechanism deepens our understanding of technology’s role in education, transcending a simple tool-based perspective.

The instructional design subsystem revolves around elements such as course objectives, teaching activities, learning resources, and assessment methods, exploring the dynamic influence between instructional design quality and learning outcomes. Well-designed GenAI-integrated instruction can elicit positive responses from learners, inspiring higher levels of engagement and reflection (R8) and providing continuous feedback for instructional optimization (R9).

The management decision subsystem focuses on factors such as GenAI promotion policies, supporting measures, and ethical governance. Resource investment and incentive mechanisms can form positive feedback driving GenAI application (R10), while ethical institutional constraints provide negative feedback regulation against technology misuse (B3). The evaluation of policy implementation effects provides a basis for management decision optimization, forming a double-loop feedback structure for policy learning.

The social environment subsystem involves external stakeholders in the educational ecosystem, such as parents, employers, and the general public. The construction of this subsystem draws on ecosystem theory, reflected in mechanisms where stakeholders indirectly shape the GenAI development environment by influencing educational policies, resource allocation, and ethical norm formulation (R11, B4). Simultaneously, the educational performance and ethical consequences produced by GenAI applications alter stakeholders’ attitudes and actions (R12), a dynamic process profoundly reflecting the complex interactive relationships among technology, education, and society.

#### 3.3.2. Stock-Flow Model Development

Based on the overall CLD, this study further developed a stock-flow model of the GenAI-supported higher education language-learning system. This model not only focuses on key condition variables identified by QCA, such as technology acceptance, learning design quality, cognitive load level, human–computer interaction quality, and ethical considerations, but also incorporates important intermediate variables and feedback structures revealed by causal loop analysis, thereby constructing a more comprehensive and dynamic system representation.

The stock-flow model framework of the GenAI-supported higher education language-learning system consists of six sectors: learner, teacher, technology, teaching, management, and environment. Each sector contains multiple stocks, flows, and auxiliary variables, as well as complex causal relationships among them. This multi-sector model structure not only reflects the system’s complexity but also provides us with a holistic perspective to understand the application dynamics of GenAI in higher education language learning.

In the learner sector, the language proficiency stock is influenced by both language-learning inflow and skill depreciation outflow, while the language-learning rate is jointly determined by factors such as learning motivation level, metacognitive strategy application efficiency, and cognitive load optimization degree. Although GenAI-supported personalized learning paths and immediate feedback can significantly improve learning efficiency, they may also introduce additional cognitive load [63]. This contradiction reflects the complexity of technology-assisted learning, necessitating a balance between efficiency improvement and cognitive load.

The teacher sector precisely depicts the dynamic change process of teachers’ GenAI adoption willingness stock. This stock is influenced by both adoption pressure (from policy incentives and student demands) and resistance (stemming from ethical concerns and capability gaps). Teachers’ willingness to use and implementation ability jointly determine the probability and intensity of GenAI teaching behaviors [64].

The technology sector models the dynamic evolution process of GenAI system performance. The technological maturity stock is affected by R&D investment inflow and technology obsolescence outflow, while perceived ease of use and usefulness determine initial adoption rates and continued use stickiness, respectively. This mechanism not only embodies the core ideas of Davis’s [59] Technology Acceptance Model but also reflects the key role of user experience in technology adoption. System reliability and interaction quality are jointly influenced by technological maturity and data accumulation [2], deepening our understanding of the continuous optimization mechanisms of AI systems in practical applications.

The teaching sector revolves around the core concept of instructional design quality. The clarity of course objectives, the match of learning activities, the appropriateness of GenAI resources, and the reasonableness of assessment schemes collectively determine the level of instructional design quality. Design quality shapes learning outcomes by influencing intermediate variables such as learning experience, teaching effectiveness, and continuous optimization [65].

The management sector focuses on the optimization process of the GenAI promotion policy stock. Policy formulation is driven by the gap between expected and actual performance, while policy implementation depends on the intensity of resource investment and the level of stakeholder cooperation, reflecting the complexity of policy implementation. Policy effect evaluation provides feedback for new rounds of policy formulation, forming a closed loop for strategy adjustment [66].

The environment sector depicts the dynamic change process of the social support stock. The educational benefits’ improvement and ethical risk control brought by GenAI applications jointly influence public attitudes, thereby changing social support levels. The level of support, in turn, affects the sustainable development of GenAI by influencing resource allocation and ethical regulation. This feedback mechanism deepens our understanding of the complex interactive relationships among technology, education, and society.

The model’s parameter estimation and calibration process comprehensively utilized methods such as reference mode fitting, partial model testing, and extreme condition testing [44] to verify structural validity and behavioral reasonableness. For parameter uncertainty, this study employed Monte Carlo simulation to conduct sensitivity analysis [67], which can help us understand the model’s sensitivity to parameter changes, providing more robust decision support for policy formulation.

## 4. Empirical Results’ Analysis of Dynamic Mechanisms of GenAI in English Language Learning

### 4.1. QCA Findings

#### 4.1.1. Single-Condition Necessity Analysis

To comprehensively understand the influence mechanisms of GenAI applications in higher education English teaching, we first conducted a necessity analysis for each condition included in the QCA. Necessity analysis aims to determine whether a condition is a necessary prerequisite for a specific outcome, i.e., whether the presence of the condition is necessary for the occurrence of the result [68].

By calculating the consistency and coverage indicators between each condition and high or low learning outcomes, we can systematically evaluate the degree of necessity of different factors [69]. Consistency measures the sufficiency relationship between conditions and outcomes, i.e., to what extent the presence of the condition ensures the occurrence of the outcome; coverage reflects the explanatory power of the condition for the outcome, i.e., to what extent the presence of the condition covers the occurrence of the outcome [41]. The results of single-condition necessity analysis are shown in Table 1.

In explaining high learning outcomes (LO_High), high levels of technology acceptance (TA), learning design quality (LDQ), human–computer interaction quality (HIQ), and ethical considerations (EC), as well as low levels of cognitive load (CLL), all demonstrated strong necessity. Among these, the necessity of low cognitive load (~CLL) was most prominent, with a consistency as high as 0.9810, indicating that reasonable control of learners’ cognitive load is a key prerequisite for achieving good learning outcomes in GenAI-supported English learning. This finding corroborates the core tenets of cognitive load theory, namely that effective instructional design should strive to manage intrinsic, extraneous, and germane cognitive loads to optimize learners’ cognitive resource allocation [51].

High-quality learning design (LDQ) and sufficient ethical considerations (EC) also showed significant necessity for high learning outcomes, both with consistency reaching 0.9620. This indicates that well-designed teaching activities, learning resources, and assessment methods, as well as attention to ethical issues such as privacy protection and fairness, are important prerequisites for successful GenAI-empowered English teaching. These findings echo the advocacy of educational ethics and responsible AI education principles [1], highlighting the fundamental position of ethical care in educational transformation in the AI era.

High levels of technology acceptance (TA) and human–computer interaction quality (HIQ) were also confirmed as strong necessary conditions for high learning outcomes, with consistencies of 0.9494 and 0.9747, respectively. This implies that learners’ acceptance attitudes and willingness to use GenAI tools, as well as the user-friendly, intelligent interactive design of GenAI systems, are key factors in realizing their educational application value. This result supports the expanded application of the technology acceptance model and human–computer interaction theory in the educational field [18], emphasizing the importance of optimizing intelligent technology design oriented towards educational needs.

Notably, in explaining low learning outcomes (LO_Low), the necessity of all conditions was relatively weak, with the highest consistency for cognitive load (CLL) being only 0.7273. This suggests that the causes of ineffective GenAI applications are more complex and diverse, and single factors can hardly fully explain poor learning outcomes. This prompts us to comprehensively consider the interactions among multiple dimensions such as technology, teaching, cognition, interaction, and ethics when analyzing the performance of GenAI educational applications, and to carefully explore the combinatorial effects of different factors in specific contexts.

Another phenomenon worth discussing is the asymmetry of condition necessity. Taking cognitive load as an example, while low cognitive load is almost a sufficient and necessary condition for high learning outcomes (consistency of 0.9810), high cognitive load is not an equally necessary condition for low learning outcomes (consistency of only 0.7273). This asymmetry exists in other conditions as well. This suggests that the influence of many factors on GenAI teaching effectiveness is not a simple linear relationship but presents complex asymmetry and diversity. In-depth analysis of this asymmetric mechanism will help us more comprehensively grasp the inherent laws of GenAI application in education.

In summary, through necessity analysis of key conditions included in QCA, this study reveals the key prerequisite factors affecting the application effectiveness of GenAI in English teaching, including high levels of support for technology acceptance, learning design quality, human–computer interaction quality, and ethical considerations, as well as effective control and management of cognitive load. These findings corroborate the explanatory power of multiple theoretical perspectives at the empirical data level, providing important clues for a comprehensive understanding of the multidimensional influence mechanisms of GenAI-empowered education. Meanwhile, the asymmetry phenomenon of condition necessity also lays the foundation for subsequent analysis of the combinatorial effects of different factors.

In terms of coverage, all necessary conditions for high learning outcomes demonstrated high explanatory power (coverage all above 0.85), indicating a close connection between these factors and the realization of teaching effectiveness. In contrast, the coverage of various conditions for low learning outcomes was generally low, again confirming that a single perspective can hardly exhaust the causes of ineffective GenAI applications, urgently requiring a more comprehensive explanatory framework from multiple theoretical perspectives.

#### 4.1.2. Configurational Paths to Effective Learning Outcomes

After determining the key prerequisite factors affecting GenAI-assisted English learning effectiveness through necessity analysis, we further employed fuzzy-set Qualitative Comparative Analysis (fsQCA) to explore the combinatorial effects among these factors, aiming to identify typical causal configurations leading to high and low learning outcomes. The core of fsQCA lies in distilling regularities from multiple case comparisons, revealing causal complexity and equifinality [70].

The analysis process followed the standard analytical procedures proposed by Ragin [37]. First, raw data were calibrated into fuzzy membership scores, representing the degree of membership of cases in different condition sets. Second, consistency and coverage indicators of various conditions were used to evaluate their necessity and sufficiency. Then, a truth table was constructed to enumerate all possible causal combinations, and typical sufficient condition configurations were identified through reduction based on frequency and consistency thresholds. Finally, through Boolean algebra simplification, parsimonious expressions leading to specific outcomes were extracted, i.e., core configurational paths.

The fsQCA results of this study revealed differentiated configurational paths leading to high learning outcomes (High Learning Outcome) and low learning outcomes (Low Learning Outcome), demonstrating the causal complexity and asymmetry in influencing GenAI-assisted English learning effectiveness (see Table 2).

For high learning outcomes, two typical configurational paths emerged. The first path (Config 1) reveals that the combination of high technology acceptance (TA), high learning design quality (LDQ), low cognitive load (~CLL), high human–computer interaction quality (HIQ), and high ethical considerations (EC) is a sufficient condition for achieving excellent learning outcomes (consistency of 0.959, raw coverage of 0.918). This configuration highlights the importance of the synergistic effect of multiple key factors, especially emphasizing the crucial role of reducing learners’ cognitive load while ensuring high quality in other elements. This may be particularly applicable to helping beginners or students facing complex learning tasks to better process information.

The second high learning outcome path (Config 2) is similar to the first one, but cognitive load (CLL) changes from a core condition to a peripheral condition. This means that even with uncertain cognitive load levels, good learning outcomes can be achieved as long as technology acceptance, learning design quality, human–computer interaction quality, and ethical considerations remain at high levels (consistency of 0.959, raw coverage of 0.899). This configuration may be more suitable for learners with rich learning experiences or strong self-regulation abilities who can learn effectively under different cognitive load levels.

For low learning outcomes, the analysis also identified two main configurational paths. The first path (Config 3) reveals that the combination of low technology acceptance (~TA), low learning design quality (~LDQ), high cognitive load (CLL), low human–computer interaction quality (~HIQ), and low ethical considerations (~EC) is a sufficient condition, leading to poor learning outcomes (consistency of 0.889, raw coverage of 0.636). This finding highlights the “perfect storm” formed when multiple factors are simultaneously in unfavorable states, especially the severe obstructive effect on the learning process when high cognitive load combines with other negative factors. This situation may occur in GenAI systems with rough design, poor user experience, and lack of ethical considerations, especially when learners have resistant attitudes towards the technology.

The second low learning outcome path (Config 4) indicates that even in cases where cognitive load (CLL) and ethical considerations (EC) are unclear, the combination of low technology acceptance, low learning design quality, and low human–computer interaction quality is sufficient to lead to learning inefficiency (consistency of 0.902, raw coverage of 0.606). This finding again corroborates the fundamental position of technology acceptance, learning design, and human–computer interaction in shaping the learning experience, indicating that the absence of these three core elements is sufficient to offset the potential positive effects of other factors.

Through comprehensive analysis of the configurational paths to high and low learning outcomes, this study yielded several valuable insights. First, symmetry and asymmetry coexist among different configurations, particularly evident in the roles of cognitive load and ethical considerations. This reminds us to be wary of simplistic linear thinking when analyzing the influence mechanisms of intelligent technology empowering education, and to carefully examine the interactions among factors. Second, the core position of technology acceptance, learning design quality, and human–computer interaction quality in all configurations highlights their fundamental role in shaping the effectiveness of GenAI educational applications, aligning with the propositions of the technology acceptance model and instructional design theory. Third, the varying role of cognitive load in different configurations reflects the complexity of managing learners’ cognitive load, potentially requiring dynamic adaptation according to individual and contextual differences. Lastly, while ethical considerations are not a necessary condition in all configurations, they are crucial in achieving high learning outcomes and avoiding low learning outcomes, which is essential for developing responsible AI education systems.

#### 4.1.3. Theoretical Insights from Configuration Analysis

This study employed fs QCA, using 33 English teaching classes at the Harbin Institute of Technology as the research sample, to systematically analyze the influence mechanisms of GenAI applications in higher education language learning. By exploring the complex interactions among multidimensional factors such as technology acceptance, learning design quality, cognitive load, human–computer interaction quality, and ethical considerations, this research revealed multiple typical causal configurations leading to high and low learning outcomes, providing empirical evidence and theoretical insights for in-depth understanding of the inherent laws of intelligent technology empowering education.

The findings highlight the multi-causal path dependence characteristics of GenAI-assisted language-learning effectiveness, corroborating the widespread consensus on the complexity of educational contexts in previous studies [71]. The realization of high learning outcomes is neither a linear result of a single element nor a random product, but the result of dynamic balance and interaction among multiple factors in specific contexts. This finding challenges the traditional reductionist view of educational technology, revealing the significant manifestation of emergent properties where “the whole is greater than the sum of its parts” in intelligent technology educational applications [72]. It implies that understanding and optimizing the effectiveness of GenAI-empowered language teaching requires transcending the limitations of a single perspective, comprehensively considering multiple dimensions such as technology, teaching, cognition, interaction, and ethics, and dynamically grasping the interactive effects, compensatory relationships, and context dependencies of various elements.

Specifically, the fsQCA results highlight the ecological concept of interaction and symbiosis among learners, intelligent systems, and teaching environments. The realization of high learning outcomes depends on learners’ high acceptance of intelligent technology, positive experiences during use, and sensitivity to ethical risks, as well as the fine-tuning of intelligent systems in functional design, interactive interfaces, and algorithmic ethics, and the careful design of teaching activities in goal guidance, content presentation, and assessment feedback. These three aspects are indispensable and collectively shape the key ecological factors supporting language learning. This finding aligns with the socio-technical systems theory of AI educational applications [73], emphasizing the co-adaptation mechanism of users, technology, and environment, providing a systematic framework for creating GenAI-based intelligent language-learning ecosystems.

This study also revealed the asymmetric characteristics of dominant factor configurations in different learning outcome contexts, highlighting the dynamic complexity of GenAI educational application mechanisms. Taking cognitive load as an example, in some high learning outcome paths, reducing cognitive load is key to achieving excellent learning outcomes, while in others, even with uncertain cognitive load levels, positive combinations of other factors can ensure learning effectiveness. This suggests that for different learners in various learning task contexts, cognitive load regulation strategies need to be dynamically adjusted. This insight corroborates the multidimensional interpretation of cognitive load theory [51], revealing the interactive shaping mechanisms of intrinsic, extraneous, and germane cognitive loads in different contexts, providing theoretical references for designing personalized intelligent learning support.

The configurational analysis also highlighted the fundamental position of ethical considerations in GenAI educational applications. The realization of high learning outcomes must be premised on effective management of ethical risks, while insufficient ethical considerations are an important inducement for low learning outcomes. This finding echoes the research consensus on educational AI ethics [1], namely that ethical awareness and capacity building should become an endogenous requirement throughout the design, development, and application process of intelligent education systems, supporting truly individualized and precise education. This means that while fully exploring the potential of GenAI technology, educators need to maintain ethical sensitivity at all times, internalizing risk awareness in all aspects of teaching practice. Only by implementing ethical principles such as promoting educational equity, protecting privacy and security, improving data quality, and optimizing algorithmic transparency in every aspect of GenAI-supported language learning can we ensure the bottom-line fairness and long-term development of technology empowerment.

### 4.2. System Dynamic Simulation Outcomes

Our system dynamic simulation of the GenAI-supported language-learning system yielded rich insights into the complex interactions and dynamics at play. We present the results through five key visualizations, each offering a unique perspective on the system’s behavior.

#### 4.2.1. System Dynamic Overview

**Figure 2:**
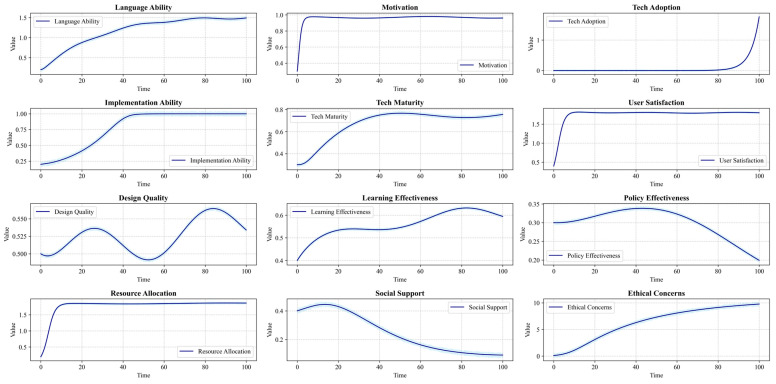
Overview simulation results of system dynamics of GenAI-supported language learning.

The temporal evolution of key system variables, as depicted in Figure 2, reveals a complex tapestry of interactions that underpin the language-learning process in a GenAI-enhanced environment. The trajectory of language ability exhibits a non-linear growth pattern characterized by periods of rapid advancement interspersed with plateaus. This observed pattern aligns remarkably well with established theories of language acquisition, such as the Dynamic Systems Theory in second language development [74]. The presence of these plateaus, or ‘silent periods’, in our simulation lends credence to the notion that language learning is not a continuous, linear process but rather a complex, dynamic system with periods of apparent stagnation that may, in fact, be crucial for the consolidation of newly acquired linguistic knowledge.

Interestingly, the motivation variable in our model displays oscillatory behavior superimposed on a generally positive trend. This finding resonates with the concept of motivational self-regulation in language learning. The observed fluctuations may represent the cyclical nature of motivation in long-term learning endeavors, where learners experience peaks and troughs in their engagement levels. The overall positive trend, however, suggests that the GenAI system plays a crucial role in sustaining and enhancing learner motivation over time, possibly through personalized feedback, adaptive challenge levels, and the novelty factor associated with AI-driven interactions.

It is important to acknowledge the potential for reverse causality in the relationships between variables in our model, particularly between learning motivation and language ability. While our simulation results suggest that higher motivation leads to improved language ability, it is plausible that the relationship is bidirectional. As learners experience success and progress in their language-learning journey, their motivation may be further enhanced, creating a positive feedback loop. Conversely, struggles or setbacks in language acquisition may dampen motivation, leading to a negative feedback loop.

Although our current model does not explicitly incorporate this reverse causality, it is essential to consider its potential impact when interpreting the simulation results. The dynamic interplay between motivation and language ability is likely more complex than a unidirectional relationship, and future research should aim to further disentangle these intricate feedback processes. By acknowledging the possibility of reverse causality, we can develop a more nuanced understanding of the factors that shape language-learning outcomes in GenAI-supported environments and guide future model refinements.

The gradual increase in technology adoption and implementation ability variables, albeit with some volatility, points to the diffusion of innovation processes in educational settings. This pattern suggests that the integration of GenAI in language learning follows a similar adoption curve to other technological innovations in education. The observed volatility could be attributed to the “implementation dip”, where initial enthusiasm is followed by challenges during the implementation phase, before eventual stabilization and improvement.

Of particular note is the behavior of the ethical concerns variable, which shows an initial increase followed by stabilization. This pattern underscores the critical importance of proactively addressing ethical issues as GenAI technology matures. Emerging technologies often face heightened scrutiny and concern before reaching a plateau of productivity. The subsequent stabilization suggests the potential development of ethical frameworks and guidelines that help mitigate these concerns over time.

#### 4.2.2. Phase Plot Analysis

**Figure 3:**
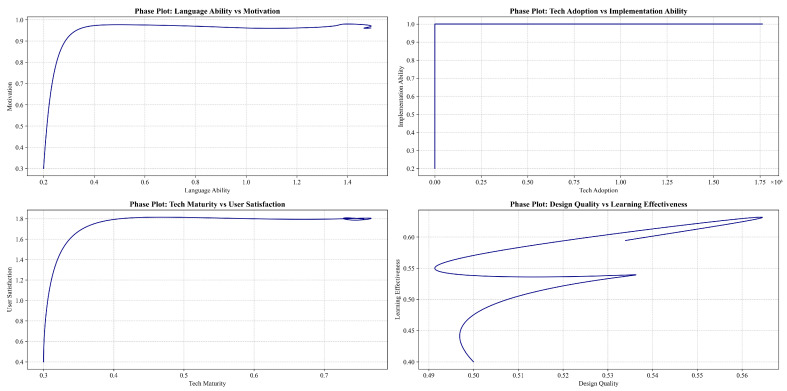
Phase plot of system dynamics of GenAI-supported language learning.

The phase plot analysis, presented in Figure 3, offers profound insights into the dyadic relationships between key system variables. The positive feedback loop observed between language ability and motivation corroborates the “Motivational Self System” theory proposed by Dörnyei (2009) [75]. This reinforcing cycle suggests that as learners perceive improvements in their language ability, their motivation increases, which, in turn, drives further language acquisition. This finding has significant implications for the design of GenAI-supported learning environments, emphasizing the importance of providing learners with clear indicators of their progress to maintain and enhance their motivation.

The relationship between technology adoption and implementation ability, as revealed in the phase plot, exhibits complex dynamics that go beyond a simple linear correlation. This pattern aligns with the Technology Acceptance Model (TAM) proposed by Davis (1989) and its subsequent extensions [17]. The observed complexity suggests that while improving implementation ability is crucial for successful technology adoption, there may be additional mediating factors at play, such as perceived usefulness and ease of use. This finding highlights the need for a multifaceted approach to promoting GenAI adoption in language-learning contexts, addressing not only the technical aspects of implementation but also user perceptions and institutional support structures.

The strong positive relationship observed between technological maturity and user satisfaction in the phase plot analysis underscores the critical role of continuous technological improvement in maintaining user engagement. This finding aligns with the Expectation-Confirmation Model in information systems research [76], suggesting that as GenAI technologies mature and meet or exceed user expectations, satisfaction levels increase, potentially leading to continued use and positive word-of-mouth dissemination.

#### 4.2.3. Correlation Heatmap

**Figure 4:**
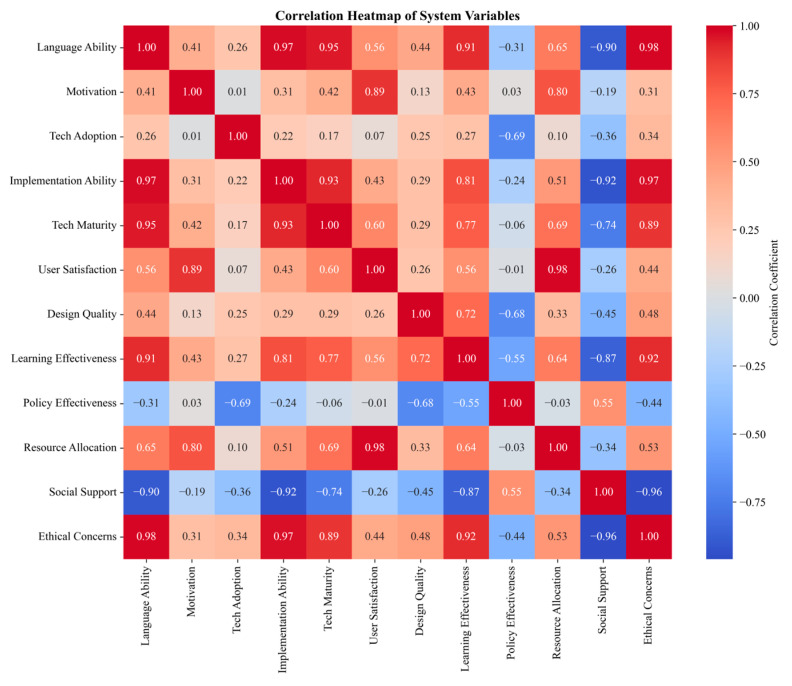
Correlation heatmap of system dynamics of GenAI-supported language learning.

The correlation analysis presented in Figure 4 provides a holistic view of the interdependencies within the GenAI-supported language-learning ecosystem. The strong positive correlations observed between language ability, motivation, and learning effectiveness validate the interconnected nature of these constructs, as posited by numerous second language acquisition theories, including Gardner’s Socio-Educational Model [77] and Dörnyei’s L2 Motivational Self System [75]. These findings suggest that GenAI systems have the potential to create virtuous cycles of improvement, where enhancements in one area (e.g., learning effectiveness) can lead to positive outcomes in others (e.g., motivation and language ability).

The moderate negative correlation between ethical concerns and technology adoption revealed in our analysis highlights a critical challenge in the widespread implementation of GenAI in language education. This finding aligns with the broader discourse on the ethical implications of AI in education, as discussed by Zawacki-Richter et al. (2019) [2]. The observed relationship underscores the necessity of addressing ethical issues transparently and proactively to foster trust and promote wider acceptance of GenAI technologies in language-learning contexts. It also points to the potential need for the development of ethical guidelines and frameworks specifically tailored to the use of AI in language education, similar to those proposed for AI in general education by organizations such as UNESCO.

The positive correlations observed between resource allocation, technological maturity, and user satisfaction emphasize the importance of sustained investment in GenAI technologies for language learning. This finding aligns with the resource-based view in strategic management, suggesting that continued allocation of resources to the development and refinement of GenAI technologies can lead to improved user satisfaction and, presumably, better learning outcomes. It also highlights the potential for a “Matthew effect” in educational technology, where institutions or programs that invest more in GenAI may see disproportionately positive results, potentially exacerbating existing inequalities in educational access and outcomes.

#### 4.2.4. Sensitivity Analysis

**Figure 5:**
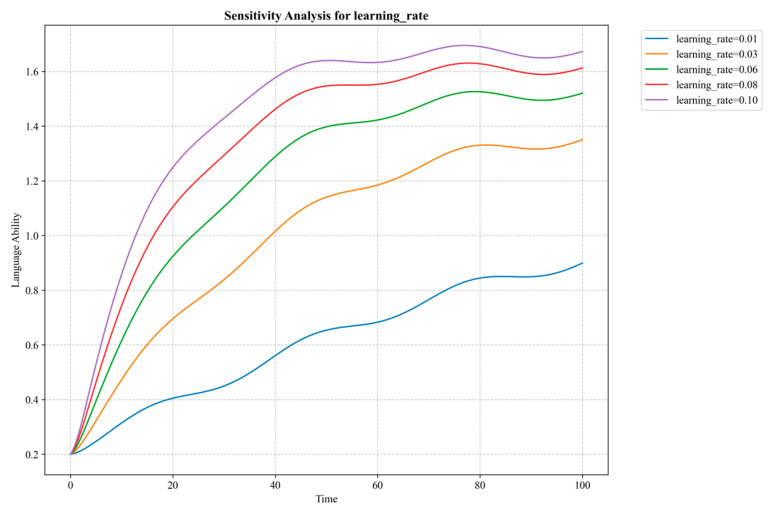
Sensitivity analysis of system dynamics of GenAI-supported language learning.

The sensitivity analysis presented in Figure 5 offers valuable insights into the system’s response to variations in the learning rate parameter. The non-linear relationship between learning rate and language ability aligns with the concept of “desirable difficulties” in learning. This finding suggests that while higher learning rates generally lead to faster language acquisition, there may be an optimal range beyond which further increases yield diminishing returns or potentially detrimental effects. The significant long-term effects observed even with small changes in the learning rate underscore the importance of fine-tuning GenAI algorithms and pedagogical approaches to optimize learning outcomes.

The resilience of the system to moderate learning rate variations, as evidenced by the overlapping confidence intervals in our sensitivity analysis, suggests a degree of robustness in GenAI-supported language-learning environments. This finding has important implications for the design and implementation of such systems, indicating that they may be able to accommodate a range of learner profiles and learning styles without significant degradation in overall performance. However, it also highlights the need for adaptive systems that can identify and respond to individual learner characteristics to maximize learning efficiency.

#### 4.2.5. Monte Carlo Simulation

**Figure 6:**
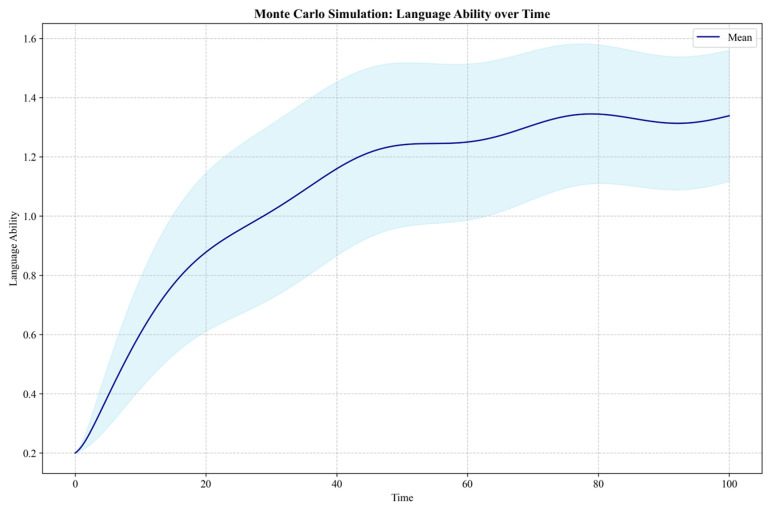
Monte Carlo simulation of system dynamics of GenAI-supported language learning.

The Monte Carlo simulation results, visualized in Figure 6, provide a probabilistic perspective on the long-term outcomes of GenAI-supported language learning. The generally upward trend in the mean trajectory of language ability, despite the introduction of stochastic elements, reinforces the potential of GenAI to positively impact language-learning outcomes. This finding aligns with meta-analyses of technology-enhanced language learning, such as those conducted by Grgurović et al. (2013) [78], which have shown overall positive effects of technology integration in language education.

The widening confidence interval observed in our Monte Carlo simulation over time reflects the increasing uncertainty inherent in long-term predictions of complex, dynamic systems. This phenomenon is well documented in chaos theory and complex system research [79] and serves as a reminder of the limitations of deterministic forecasting in educational contexts. However, the fact that even the lower bound of the confidence interval shows improvement over time is a strong indicator of the robustness of GenAI’s positive impact on language-learning outcomes.

In conclusion, our system dynamic simulation provided a comprehensive and nuanced understanding of the GenAI-supported language-learning ecosystem. The results not only corroborate existing theoretical frameworks in second language acquisition, educational technology, and motivation research, but also offer novel insights into the complex interplay of factors shaping this emerging field. The simulation highlights the potential of GenAI to create positive feedback loops in language learning while also underscoring the importance of addressing ethical concerns, optimizing learning parameters, and maintaining technological investment.

#### 4.2.6. Theoretical Correspondence Between Simulation Results and fsQCA Findings

This study employed system dynamics’ modeling to conduct an in-depth analysis of the GenAI-supported university English learning ecosystem. The results obtained strongly resonate with and cross-validate the key factor configurations influencing learning effectiveness revealed by the fsQCA presented earlier. This integration of qualitative and quantitative, static and dynamic analytical perspectives provides unique and valuable theoretical insights for comprehensively understanding the complex dynamics of intelligent technology empowering language learning.

The non-linear trajectory of language ability development revealed by system dynamic simulation forms an interesting contrast with the multiple causal paths identified by fsQCA. The simulation results show dynamic characteristics of language ability exhibiting periodic rapid progress interwoven with plateaus, which highly aligns with the dynamic balance of element combinations emphasized by fsQCA. This interplay of “static” and “dynamic” analyses corroborates the complexity and non-linearity of the language-learning process, highlighting the complementary advantages and synergistic effects of different elements at various stages of the learning process. As revealed by fsQCA, reducing cognitive load may be crucial in the early stages of learning, while technology acceptance and human–computer interaction quality may become more prominent in later stages. This suggests that when designing and implementing intelligent language-learning support, we need to dynamically adjust the proportion of various elements according to time and context, achieving real-time resonance and precise service with learners.

However, it is essential to acknowledge that while the correspondence between fsQCA findings and simulation results provides valuable insights, the observational nature of our study limits the causal claims that can be made. The associations revealed by both methods suggest intriguing patterns and relationships, but further experimental research is needed to establish definitive causal connections. By explicitly recognizing this limitation, we aim to present a more nuanced and balanced interpretation of our findings, emphasizing the need for continued research to validate and extend the insights gained from this exploratory study.

This cautionary note is particularly relevant when considering the complex interplay of factors influencing learning effectiveness in GenAI-supported environments. While our theoretical framework, grounded in the integration of the Technology Acceptance Model, Teaching Presence Theory, Cognitive Load Theory, Human–Computer Interaction Theory, and Educational Ethics Framework, provides a comprehensive lens for interpreting the results, it is plausible that other factors beyond the scope of our study may also shape learning outcomes. For instance, individual differences in learner characteristics, such as prior language proficiency, learning styles, and self-regulation strategies, could interact with the identified configurations to influence learning effectiveness. Learners with higher initial proficiency may be better equipped to navigate the challenges of GenAI-supported learning, while those with more adaptive learning styles and effective self-regulation strategies may be more likely to thrive in this innovative educational context.

Moreover, contextual factors, such as institutional support, technological infrastructure, and socio-cultural norms, may play a significant role in the successful implementation and impact of GenAI in language education. Institutions with robust technological infrastructure and dedicated support for GenAI integration may create more favorable conditions for effective learning, while those lacking these resources may face additional barriers. Similarly, socio-cultural norms surrounding the acceptance and use of AI technologies in education may influence learners’ and educators’ willingness to engage with GenAI tools, ultimately shaping the realized benefits.

By acknowledging these potential alternative explanations, we aim to provide a more robust and comprehensive analysis of the complex dynamics at play. While our study offers compelling evidence for the identified patterns and relationships, we recognize that a fuller understanding of the factors driving GenAI effectiveness in language learning requires ongoing research that considers a broader range of individual, contextual, and socio-cultural variables. This recognition underscores the importance of continued scholarly inquiry to refine and expand the theoretical models guiding the design and implementation of GenAI in educational settings.

The simulation results also capture a unique pattern of cyclical fluctuations in learning motivation superimposed on an overall upward trend. This echoes the multiple causal paths revealed by fsQCA, jointly corroborating the compound regulation mechanism of learning motivation. On the one hand, high-quality intelligent learning experiences can sustainably drive the enhancement of intrinsic learning motivation; on the other hand, external support and incentives are also necessary, such as teacher guidance and peer interaction stimulation. This combination of static and dynamic regulatory mechanisms, shaping both individual and social dimensions, resonates beautifully with many classic learning motivation theories, such as Dörnyei’s L2 Motivational Self System and Gardner’s Socio-Educational Model.

The correspondence between these simulation results and established theoretical frameworks offers robust support for the validity of our findings. Dörnyei’s L2 Motivational Self System, which emphasizes the interplay of learners’ ideal L2 self, ought-to L2 self, and L2 learning experience, provides a compelling lens for interpreting the cyclical fluctuations in motivation observed in our simulations. As learners engage with GenAI tools, their positive experiences may enhance their ideal L2 self, bolstering intrinsic motivation. However, the ought-to L2 self, shaped by external expectations and pressures, may also contribute to motivational fluctuations, as learners navigate the challenges and demands of the learning process. Similarly, Gardner’s Socio-Educational Model, which highlights the role of social and contextual factors in shaping language-learning motivation, aligns with our findings on the importance of teacher guidance and peer interaction in sustaining motivation.

This theoretical consonance underscores the value of our simulation results in providing empirical support for key tenets of language-learning motivation theory. By demonstrating the dynamic interplay of intrinsic and extrinsic factors in shaping motivational trajectories, our study contributes to the ongoing refinement and validation of these theoretical frameworks. Moreover, these insights illuminate the critical role of carefully designed human–machine collaboration and teacher–student interaction in optimizing motivation within intelligent technology-driven language-learning ecosystems. As we move forward in the development and implementation of GenAI tools, attending to these motivational dynamics will be essential for realizing the full potential of these innovative educational approaches.

Furthermore, the high consistency of ethical concerns in both simulation results and fsQCA findings is noteworthy. Ethical issues consistently shadow and determine the success or failure of intelligent technology applications in language education, which is a common feature of the high learning effectiveness paths revealed by fsQCA and is fully presented in the system dynamic simulation. Whether it is the periodic evolution of the ethical concern variable in the model or its complex association with technology acceptance, it highlights its key role as a “stabilizer” in the intelligent language-learning ecosystem.

This finding aligns with and extends relevant theoretical frameworks, such as Holmes et al.’s AI education ethics framework [1] and Aiken’s responsible AI principles [27]. Holmes et al.’s framework emphasizes the importance of considering ethical dimensions throughout the design, development, and deployment of AI technologies in education, including issues of fairness, accountability, transparency, and privacy [1]. Our simulation results provide empirical support for the centrality of these ethical considerations, demonstrating their pervasive influence on the dynamics of GenAI-supported language learning. Similarly, Aiken’s responsible AI principles underscore the need for AI systems to be robust, transparent, accountable, and aligned with human values. The prominence of ethical concerns in our findings suggests that adherence to these principles is not merely an abstract ideal but a concrete determinant of the effectiveness and sustainability of GenAI in language education.

These theoretical correspondences underscore the importance of deeply integrating ethical considerations into the development and implementation of intelligent language-teaching applications. As our simulations demonstrate, neglecting these ethical dimensions can lead to suboptimal outcomes and undermine the long-term viability of GenAI-supported learning. Conversely, proactively addressing ethical concerns and embedding them into the fabric of intelligent language-learning ecosystems can create the conditions for these innovative technologies to mature and excel. This recognition calls for a sustained commitment to interdisciplinary collaboration among language educators, AI researchers, ethicists, and policymakers to ensure that the transformative potential of GenAI in language education is realized in a responsible, equitable, and value-aligned manner.

The non-linear impact of parameter adjustments such as learning rate and initial motivation level on system behavior revealed by system dynamic sensitivity analysis also forms an interesting correspondence with fsQCA findings. This further verifies the dynamic complexity of the language-learning process, prompting us to carefully evaluate the long-term effects of different instructional designs and system optimization strategies. Flexible control of the learning rhythm in teaching practice and tailoring to learners’ characteristics and needs are crucial for realizing the long-term educational value of intelligent language-teaching applications.

This insight resonates with the intrinsic essence of adaptive learning theory and personalized education concepts. Adaptive learning theory posits that optimal learning occurs when the learning experience is dynamically adjusted to match the evolving needs, abilities, and characteristics of individual learners. Our sensitivity analysis results provide compelling evidence for the importance of this adaptability in the context of GenAI-supported language learning. By demonstrating the non-linear effects of parameter variations on learning outcomes, our study highlights the need for intelligent language-learning systems that can flexibly adjust to learners’ diverse profiles and progressions.

Similarly, personalized education concepts emphasize the tailoring of learning experiences to individual learners’ strengths, interests, and goals. The correspondence between our sensitivity analysis findings and fsQCA results underscores the value of this personalization in the realm of GenAI-supported language learning. As our simulations demonstrate, one-size-fits-all approaches are likely to yield suboptimal outcomes, given the complex interplay of factors influencing learning effectiveness. Instead, realizing the full potential of GenAI in language education requires a commitment to learner-centered design, in which intelligent systems are optimized to adapt to the unique needs and trajectories of each language learner.

These theoretical alignments highlight the transformative potential of GenAI in reshaping language education towards more adaptive, personalized, and equitable learning experiences. By harnessing the power of intelligent technologies to dynamically adjust learning rhythms, instructional strategies, and support mechanisms, we can create language-learning ecosystems that meet learners where they are and empower them to achieve their full potential. However, realizing this vision will require sustained collaboration among researchers, educators, and technology developers to refine the design and implementation of GenAI systems in light of these theoretical insights. As we continue to explore the frontiers of intelligent language learning, a steadfast commitment to theory-driven, learner-centered innovation will be essential for unlocking the transformative potential of GenAI in language education.

In interpreting these findings, it is important to consider the limitations inherent in our observational study design. While the convergence of fsQCA and system dynamic results provides compelling evidence for the identified patterns and relationships, we must be cautious in making definitive causal claims. The associations revealed by our analyses suggest promising avenues for future research, but experimental studies are needed to rigorously test and validate these causal hypotheses. By transparently discussing the limitations of our causal inferences, we aim to provide a balanced and rigorous presentation of our findings, inviting further research to build upon and extend the insights gained from this exploratory study.

Moreover, it is important to acknowledge that our study, while comprehensive in its theoretical scope and methodological approach, represents a snapshot of a rapidly evolving field. As GenAI technologies continue to advance and mature, new challenges and opportunities for language education will undoubtedly emerge. The theoretical correspondences and practical implications identified in our study should be viewed as a foundation for ongoing research and refinement, rather than a definitive endpoint. As the field of GenAI-supported language learning continues to evolve, it will be essential for researchers and educators to remain attuned to emerging developments, adapting theoretical frameworks and pedagogical practices to keep pace with technological change.

In conclusion, the theoretical correspondence between our system dynamic simulation results and fsQCA findings offers a powerful lens for understanding the complex dynamics of GenAI-supported language learning. By integrating qualitative and quantitative, static and dynamic perspectives, our study provides a comprehensive and nuanced understanding of the factors shaping learning effectiveness in intelligent language-learning ecosystems. While the observational nature of our study limits the causal claims that can be made, the compelling patterns and relationships revealed by our analyses provide a solid foundation for future research and practice.

As we move forward in the development and implementation of GenAI technologies in language education, it will be essential to remain grounded in robust theoretical frameworks while also attending to the ethical, contextual, and individual factors that shape learning outcomes. By embracing a commitment to theory-driven, learner-centered innovation, we can harness the transformative potential of GenAI to create more adaptive, personalized, and equitable language-learning experiences for all. The insights and implications of our study offer a roadmap for this exciting and challenging journey, inviting researchers, educators, and technology developers to collaborate in shaping the future of intelligent language learning.

## 5. Discussion

### 5.1. Research Summary

This study focused on 33 English teaching classes at the Harbin Institute of Technology, employing a mixed research paradigm combining QCA and SD simulation to systematically investigate the influence mechanisms of GenAI applications in university English teaching. Based on theoretical perspectives including the Technology Acceptance Model, Teaching Presence Theory, Cognitive Load Theory, Human–Computer Interaction Theory, and Educational Ethics Framework, we constructed an integrative theoretical framework encompassing six subsystems: learner, teacher, technology, teaching, management, and environment. On this foundation, we proposed a series of theoretical hypotheses regarding English learning effectiveness under GenAI support: High technology acceptance, high learning design quality, low cognitive load, high human–computer interaction quality, and high ethical considerations are necessary prerequisites for achieving high learning effectiveness in GenAI-supported English teaching. Specific configurations of high technology acceptance, high learning design quality, low cognitive load, high human–computer interaction quality, and high ethical considerations are sufficient for achieving high learning effectiveness in GenAI-supported English teaching. Specific configurations of low technology acceptance, low learning design quality, high cognitive load, low human–computer interaction quality, and low ethical considerations are sufficient for leading to low learning effectiveness in GenAI-supported English teaching. These hypotheses were operationalized by linking them to specific, measurable outcomes, such as students’ language proficiency scores, engagement levels, and satisfaction ratings, as well as teachers’ perceptions of effectiveness and efficiency in GenAI-supported teaching.

At the empirical research level, we first employed the QCA method to systematically examine the complex relationships between key elements in five dimensions—learner, teacher, technology, teaching, and environment—and learning outcomes. Through necessary condition analysis, we found that high technology acceptance, high learning design quality, low cognitive load, high human–computer interaction quality, and high ethical considerations are necessary prerequisites for achieving high learning effectiveness. Building on this, we further conducted sufficient condition analysis, identifying two key paths leading to high learning effectiveness: “high technology acceptance × high learning design quality × low cognitive load × high human–computer interaction quality × high ethical considerations” and “high technology acceptance × high learning design quality × high human–computer interaction quality × high ethical considerations”. Simultaneously, we discovered two typical paths resulting in low learning effectiveness: “low technology acceptance × low learning design quality × high cognitive load × low human–computer interaction quality × low ethical considerations” and “low technology acceptance × low learning design quality × low human–computer interaction quality”. These findings not only validated the appropriateness of our initial theoretical framework but also provided targeted guidance for optimizing GenAI applications in English teaching practices.

Our findings align with and extend previous research on the factors influencing the effectiveness of educational technology applications. For instance, our results corroborate the importance of technology acceptance, learning design quality, and human–computer interaction quality in shaping learning outcomes, as highlighted in prior studies. However, our study goes beyond these singular factors by revealing their combinatorial effects and necessary and sufficient conditions for high and low effectiveness, offering a more nuanced understanding of their complex interplay in the context of GenAI-supported English teaching.

One unexpected finding was the prominence of ethical considerations as a key factor in both necessary and sufficient conditions for high learning effectiveness. While the role of ethics in AI applications has been increasingly discussed, its specific impact on learning outcomes in GenAI-supported education has been less explored. Our study highlights the critical importance of addressing ethical issues, such as privacy, fairness, and transparency, in the design and implementation of GenAI systems for English teaching. This finding suggests that ethical considerations should be given more attention in future research and practice on educational AI applications.

To further reveal the dynamic evolutionary patterns of GenAI-empowered English learning, we constructed a system dynamics simulation model encompassing six subsystems and multiple feedback loops based on the QCA results. The simulation results showed that students’ language ability development exhibits non-linear, stage-wise dynamic characteristics, aligning with the interactionist theory of language acquisition. This finding supports and extends previous research on the dynamic and complex nature of language-learning processes, highlighting the potential of GenAI systems to capture and adapt to these nonlinear patterns.

Learning motivation displayed a compound pattern of cyclical fluctuations superimposed on an overall upward trend, corroborating the multidimensional regulatory mechanism of motivation. This finding suggests that GenAI systems should be designed to not only sustain but also enhance learner motivation over time, by providing personalized support, adaptive challenges, and engaging interactions. Additionally, the simulation captured complex interactions between ethical concerns and technology acceptance, reemphasizing the foundational role of ethical considerations in intelligent language-teaching systems. This finding resonates with recent calls for responsible and human-centered AI development, underlining the need for integrating ethical principles into the design and deployment of GenAI technologies in education.

Integrating the key influential factor configurations identified by QCA with the dynamic evolutionary patterns revealed by SD, this study comprehensively depicts the multidimensional influence mechanisms and dynamic generative logic of GenAI applications in university English teaching. On the one hand, the co-adaptation of learners, intelligent systems, and teaching environments is the core essence of achieving ideal learning outcomes, with the combinatorial effects of multiple elements in specific contexts being crucial in shaping learning experiences and effects. This insight aligns with the ecological perspective on language learning, highlighting the importance of considering the complex interactions between learners, technologies, and contexts in designing effective GenAI-supported learning environments.

On the other hand, the efficacy of GenAI-empowered English learning exhibits complex characteristics such as stage-wise, cyclical, and non-linear features in its dynamic evolution, with the dynamic balance and real-time regulation of different influential factors being key to ensuring the long-term operation of intelligent teaching systems. This finding extends the understanding of educational AI applications from a static, one-shot view to a dynamic, lifecycle perspective, emphasizing the need for continuous monitoring, adaptation, and optimization of GenAI systems over time. It also highlights the potential of system dynamic modeling as a powerful tool for understanding and managing the complexity of intelligent language-learning ecosystems.

It can be said that the theoretical findings obtained through the mixed paradigm of QCA and SD in this study not only enrich the research perspective of intelligent language education but also lay a methodological foundation for exploring educational AI applications. On the one hand, the element configurations revealed by qualitative analysis reflect the generative logic of intelligent teaching activities, inspiring us to systematically design derivative teaching schemes from multidimensional perspectives and contextual characteristics, tailoring education and implementing precise strategies. On the other hand, the dynamic evolutionary patterns depicted by quantitative modeling provide a unique perspective for grasping the emergent properties and long-term effects of intelligent teaching systems, guiding us to continuously enhance teaching experiences and educational quality through dynamic assessment and real-time optimization.

### 5.2. Theoretical Contributions

This study makes several important theoretical contributions to the field of intelligent language education and educational technology research. Firstly, the integrative theoretical framework constructed in this study achieves a systematic fusion of multidisciplinary perspectives, addressing the first research gap identified in the introduction regarding the lack of comprehensive examination of factors influencing GenAI effectiveness. By considering six key dimensions (learner, teacher, technology, teaching, management, and environment) and drawing on theories from diverse fields such as technology acceptance, teaching presence, cognitive load, human–computer interaction, and educational ethics, our framework provides a holistic and nuanced lens for understanding the complex interplay of elements shaping GenAI applications in English teaching. This multidimensional framework expands the theoretical boundaries of intelligent language education research, moving beyond the linear and fragmented analysis of single factors to capture the dynamic and contextual nature of GenAI-supported learning.

Secondly, the application of QCA in this study offers a novel methodological approach to unraveling the generative mechanisms of GenAI effectiveness, further contributing to filling the first research gap. By systematically examining the combinatorial effects and necessary and sufficient conditions of key elements across different dimensions, QCA enables the identification of multiple pathways to high and low learning effectiveness in GenAI-supported English teaching. This configurational perspective challenges the prevailing assumptions of unifinality and linear causality in educational technology research, revealing the complex and context-dependent nature of GenAI success. Our QCA findings provide a new theoretical vocabulary and analytical tool for understanding the “chemical reactions” and “ecological niches” of intelligent language learning, opening up fresh possibilities for theory development and empirical investigation in this field.

Thirdly, the pioneering use of SD modeling in this study addresses the second research gap concerning the insufficient consideration of the long-term and dynamic nature of GenAI applications. By conceptualizing GenAI-supported English learning as a complex system with multiple interacting elements and feedback loops, SD enables the mapping and simulation of the evolutionary trajectories and emergent patterns of key variables such as language ability, learning motivation, and ethical concerns over time. This dynamic perspective extends the theoretical horizon of intelligent language education research, moving beyond the static and cross-sectional analysis of GenAI effectiveness to capture the temporal and adaptive dimensions of technology-enhanced learning. Our SD findings provide a new theoretical framework and methodological approach for understanding the generative mechanisms and long-term impacts of GenAI interventions, contributing to the development of process-oriented and sustainable theories of intelligent language education.

Fourthly, the innovative integration of QCA and SD in a mixed-methods research design represents a significant methodological advancement in educational technology research. By leveraging the strengths of qualitative and quantitative, static and dynamic, variable-oriented and case-oriented approaches, this study provides a more comprehensive and rigorous examination of GenAI applications in English teaching. The cross-fertilization of QCA and SD, where QCA findings inform the conceptualization and parameterization of SD models, and SD simulations enrich the interpretation and extrapolation of QCA results, demonstrates the power of methodological synergy in addressing complex educational phenomena. This mixed-methods approach not only enhances the validity and reliability of our findings but also sets a new standard for multi-method inquiry in intelligent language education research.

Finally, the theoretical and methodological innovations of this study have important implications for addressing the research gaps and advancing the knowledge frontiers in the broader field of educational technology. The integrative framework, QCA and SD methods, and mixed-methods design developed in this study can serve as a template and toolkit for investigating other educational AI applications beyond the context of English teaching. By providing a holistic, configurational, dynamic, and multi-method perspective on the design, implementation, and evaluation of intelligent tutoring systems, adaptive learning platforms, and learning analytics tools, this study contributes to the theoretical and methodological foundations of smart education research. The insights and approaches generated from this study can inform the development of more comprehensive, context-sensitive, and sustainable theories and models of technology-enhanced learning, as well as guide the empirical investigation and practical application of educational AI in diverse domains.

In conclusion, this study makes significant theoretical and methodological contributions to the field of intelligent language education and educational technology research. By constructing an integrative framework, applying QCA and SD methods, and employing a mixed-methods design, this study addresses the key research gaps identified in the introduction and advances the understanding of the complex, dynamic, and generative nature of GenAI applications in English teaching. The theoretical and methodological innovations of this study not only enrich the knowledge base and analytical toolkit of intelligent language education research but also have important implications for the broader field of educational technology. As educational AI continues to evolve and transform learning and teaching processes, the insights and approaches generated from this study can provide a valuable foundation and roadmap for future research and practice in this exciting and challenging field.

### 5.3. Practical Implications

The findings of this study have significant practical implications for optimizing GenAI applications in university English teaching. Based on the key influential factor configurations and dynamic evolutionary patterns identified, we propose the following recommendations for educators, policy-makers, and technology developers:

① Adopt a holistic and integrative approach to the design and implementation of GenAI systems in English teaching. Rather than focusing on singular factors, such as technology features or pedagogical strategies, it is crucial to consider the multidimensional interplay of learner, teacher, technology, teaching, management, and environmental elements. This requires close collaboration and communication among different stakeholders to ensure the alignment and synergy of various components in creating an optimal intelligent learning ecosystem.
② Pay close attention to the necessary conditions for high learning effectiveness, such as high technology acceptance, high learning design quality, low cognitive load, high human–computer interaction quality, and high ethical considerations. These factors should serve as essential design principles and evaluation criteria for GenAI applications in English teaching. For instance, educators should carefully select and adapt GenAI tools that are user-friendly, pedagogically sound, cognitively manageable, and ethically responsible. Policy-makers should provide guidelines and resources to support the development and adoption of GenAI systems that meet these criteria.
③ Leverage the sufficient paths to high learning effectiveness identified in this study to guide the configuration and customization of GenAI applications in different teaching contexts. For example, in settings where learners have high technology acceptance and teachers can ensure high learning design quality, GenAI tools with advanced features and adaptive capabilities can be deployed to maximize learning outcomes. In contrast, in contexts where learners have low technology readiness or teachers face challenges in instructional design, GenAI applications should be introduced more gradually and with stronger scaffolding and support.
④ Monitor and address the dynamic evolutionary patterns of key elements in GenAI-supported English learning, such as students’ language ability, learning motivation, and ethical concerns. This requires establishing a continuous assessment and feedback loop to track the performance and experience of learners and teachers over time, and making timely adjustments and interventions based on the data. For instance, if students’ learning motivation shows a cyclical fluctuation pattern, teachers can proactively design engaging activities and provide personalized feedback to sustain and enhance their interest and persistence.
⑤ Foster a supportive and agile organizational environment for implementing GenAI innovations in English teaching. This includes providing adequate technical infrastructure, professional development opportunities for teachers, and incentives for experimentation and improvement. Moreover, it is essential to establish clear policies and guidelines for the ethical and responsible use of GenAI technologies, such as data privacy protection, algorithmic fairness, and human oversight. Regular communication and collaboration among administrators, teachers, students, and technology providers should be encouraged to ensure the smooth and effective integration of GenAI in teaching practices.
⑥ Conduct ongoing research and evaluation to refine and optimize GenAI applications in English teaching. As the field of educational AI is rapidly evolving, it is important to keep abreast of the latest developments and best practices, and continuously iterate and improve the design and implementation of GenAI systems based on empirical evidence and feedback from stakeholders. This study provides a comprehensive framework and methodology for such research and evaluation efforts, which can be adapted and extended in different contexts.

## 6. Conclusions

### 6.1. Main Conclusions

This study, focusing on 33 English teaching classes at the Harbin Institute of Technology, employed a mixed research paradigm combining QCA and SD simulation to explore the multidimensional influence mechanisms and dynamic evolutionary patterns of GenAI applications in university English teaching in depth. This research addressed two core issues raised in the introduction: theoretical interpretation and empirical analysis of GenAI-empowered language teaching. Based on a solid theoretical foundation and innovative methodological design, this study achieved a series of original findings.

Firstly, through a systematic review of interdisciplinary research, this study constructed an integrative analytical framework encompassing six dimensions: learner, teacher, technology, teaching, management, and environment. This framework aligns with the concept of multidisciplinary theoretical integration proposed in the research design, achieving a systematic analysis of key elements in intelligent language teaching and expanding the theoretical connotations of smart education. This finding responds to the need for understanding the mechanisms of intelligent technology empowering language learning as stated in the introduction.

Secondly, QCA analysis guided by the framework revealed two typical element combination paths for high and low effectiveness in GenAI-empowered English teaching, highlighting the decisive role of dynamic balance among learner, teacher, technology, teaching, and environmental elements in intelligent teaching effectiveness. This finding deepens the fragmented examination of factors influencing GenAI educational applications in existing research, emphasizing the importance of integrating multidimensional perspectives and validating the necessity of employing the QCA method as proposed in the research design.

Thirdly, SD simulation results vividly depicted the evolutionary landscape of the GenAI-driven English learning system, revealing stage-wise, cyclical, and non-linear evolutionary characteristics of key elements such as learners’ language ability, learning motivation, and ethical concerns, as well as their dynamic feedback mechanisms with teacher and environmental elements. This finding addresses the lack of dynamic and long-term considerations in previous studies on GenAI educational applications, validating the concept of using SD modeling to grasp the evolutionary patterns of intelligent teaching ecosystems as proposed in the introduction.

Synthesizing these empirical findings, this study systematically revealed the “multidimensional-dynamic-generative” influence mechanism of GenAI empowering university English teaching. This mechanism involves the multidimensional interaction of intelligent technology, teacher practices, learner factors, and institutional ethical environments, continuously generating new teaching paradigms, learning models, and educational pathways through the process of dynamically achieving balance and breakthroughs. This theoretical insight largely responds to and surpasses the research demands raised in the introduction, also reflecting the unique advantages of the “static QCA-dynamic SD” integrated paradigm proposed in the research design.

However, it is crucial to acknowledge that while GenAI technologies hold immense potential for transforming language education, their current capabilities and limitations must be critically assessed to ensure practical relevance and theoretical grounding. While this study has highlighted the multifaceted benefits and applications of GenAI in English teaching, it is essential to recognize that existing GenAI systems are still in their nascent stages of development and integration within educational contexts. The effectiveness and adaptability of GenAI technologies in addressing the diverse needs of language learners, particularly those with varying proficiency levels, learning styles, and cultural backgrounds, require further investigation and refinement. Moreover, the ethical implications of deploying GenAI systems in educational settings, such as issues of privacy, bias, and transparency, necessitate ongoing scrutiny and the development of robust ethical frameworks to guide their responsible implementation.

Furthermore, the successful integration of GenAI in language education relies heavily on the readiness and competence of teachers in leveraging these technologies effectively. The current state of teacher professional development and support in the context of GenAI adoption warrants additional attention and research to ensure that educators are adequately prepared to harness the potential of these tools while navigating their limitations.

### 6.2. Limitations and Future Research Directions

It is also important to recognize that this study, while offering valuable insights, has certain limitations that should be acknowledged and addressed in future research endeavors. The sample size, while diverse within the context of the Harbin Institute of Technology, may limit the generalizability of the findings to other educational institutions and contexts. Expanding the research scope to include a larger sample of classes across multiple universities and disciplines would enhance the external validity and applicability of the results. Additionally, while this study’s theoretical framework encompassed six key dimensions, there may be other pertinent factors, such as learner motivation, self-regulation, and socio-cultural influences, that could further enrich the understanding of GenAI’s impact on language learning. Incorporating these elements in future studies would provide a more comprehensive view of the complex interplay between GenAI and language education. Methodologically, the integration of QCA and SD simulation offers a powerful approach to unraveling the multidimensional and dynamic aspects of GenAI-empowered teaching. However, the inclusion of qualitative data, such as interviews with students, teachers, and administrators, could provide deeper insights into the experiential aspects of GenAI adoption and help triangulate the findings from the quantitative analyses.

Future research directions should focus on addressing these limitations and expanding the scope of inquiry. Conducting longitudinal studies to track the long-term impact of GenAI on language-learning outcomes, as well as exploring the differential effects of GenAI across various learner subgroups, would provide valuable insights for tailoring these technologies to specific educational needs. Investigating the role of teacher professional development in facilitating effective GenAI integration and examining the institutional and policy factors that enable or hinder GenAI adoption in language education are also critical avenues for future research.

In conclusion, this study represents a significant step forward in understanding the multidimensional influence mechanisms and dynamic evolutionary patterns of GenAI applications in university English teaching. By integrating QCA and SD simulation, this research has revealed the complex interplay of learner, teacher, technology, teaching, management, and environmental factors in shaping the effectiveness of GenAI-empowered language education. While this study has its limitations, it lays a solid foundation for future research and practice in this rapidly evolving field.

As GenAI technologies continue to advance and permeate language classrooms, it is crucial for researchers, educators, and policymakers to engage in ongoing dialogue and collaboration to harness their potential while addressing the challenges and ethical considerations that arise. By critically examining the current state and limitations of GenAI in education, this study contributes to a more nuanced and balanced understanding of its transformative potential and the work that lies ahead in realizing its full promise for language teaching and learning.

## Figures and Tables

**Table 1:** Analysis of the necessary conditions.

Condition	LO_High		LO_Low	
	Cons_High	Cov_High	Cons_Low	Cov_Low
TA	0.9494	0.8630	0.6667	0.1481
~TA	0.2911	0.4674	0.454545	0.168067
LDQ	0.9620	0.8621	0.727273	0.173913
~LDQ	0.2785	0.4674	0.454545	0.168067
CLL	0.2658	0.4674	0.727273	0.170732
~CLL	0.9810	0.8714	0.454545	0.171429
HIQ	0.9747	0.8506	0.772727	0.188406
~HIQ	0.2658	0.4889	0.409091	0.148148
EC	0.9620	0.8621	0.727273	0.173913
~EC	0.2785	0.4674	0.454545	0.168067

~ means there is no such variable.

**Table 2:** Configuration for high and low learning effectiveness of GenAI-assisted English learning.

Condition	High Learning Outcome		Low Learning Outcome	
	Config 1	Config 2	Config 3	Config 4
TA	●	●	○	○
LDQ	●	●	○	○
CLL	○	⊗	●	⊗
HIQ	●	●	○	○
EC	●	●	○	⊗
Consistency	0.959	0.959	0.889	0.902
Raw coverage	0.918	0.899	0.636	0.606
Unique coverage	0.019	0.000	0.030	0.000
Solution consistency	0.959		0.889	
Solution coverage	0.918		0.667	

● Indicates core condition (present); ⊗ indicates core condition (absent); ○ indicates a marginal condition (present).

## Data Availability

This article encompasses this study’s original contributions; further inquiries can be addressed to the corresponding author.
